# A Ponatinib-Associated Transcriptomic Signature: Implications for Cardiovascular Toxicity

**DOI:** 10.3390/ijms27094058

**Published:** 2026-04-30

**Authors:** Joonho Kong, Jaeyeon Jang, Jee Hyun Kong, Taesic Lee

**Affiliations:** 1 Department of Medicine, Yonsei University Wonju College of Medicine, Wonju 26426, Republic of Korea; gasese1@naver.com; 2 Division of Hematology-Oncology, Department of Internal Medicine, Yonsei University Wonju College of Medicine, Wonju 26426, Republic of Korea; jaeyeon0136@gmail.com; 3 Department of Convergence Medicine, Yonsei University Wonju College of Medicine, Wonju 26426, Republic of Korea

**Keywords:** ponatinib, cardiovascular toxicity, transcriptomic signature, iPSC-derived cardiomyocytes, meta-analysis, tyrosine kinase inhibitor

## Abstract

Ponatinib, a third-generation BCR::ABL1 inhibitor, has antileukemic activity but is associated with cardiovascular toxicity, for which transcriptome-level responses remain incompletely characterized. Here, we defined a ponatinib-associated transcriptomic signature and examined its mechanistic implications using two public RNA sequencing (RNA-Seq) datasets: GSE186341 (11 cancer cell lines treated with kinase inhibitors) and GSE217421 (induced pluripotent stem cell (iPSC)-derived cardiomyocytes treated with approved drugs). Principal component analysis (PCA) and *k*-means clustering were used to define expression-based subgroups of vehicle-treated (DMSO) controls. DESeq2, followed by fixed-effect meta-analysis, estimated subgroup-specific treatment effects and pooled effect estimates across subgroups. In GSE186341, we identified 2639 meta-analytic differentially expressed genes (meta-DEGs). Among these, 81 genes were also differentially expressed in GSE217421 after ponatinib treatment, identifying an overlapping gene set across datasets. In contrast, imatinib showed no overlap with these 81 genes under the same cross-dataset analysis framework. Cardiotoxicity-relevant functions were represented by directionally consistent genes linked to cardiac repolarization-associated ion handling (*KCNN3*), insulin-responsive metabolic regulation (*FOXO1*, *HK2*), cyclic adenosine monophosphate (cAMP)-responsive stress signaling (*RAPGEF3*), and mitochondrial homeostasis and redox regulation (*MCL1*, *GCH1*). Collectively, these results define a ponatinib-associated transcriptomic signature and nominate cross-dataset transcript-level candidates for subsequent mechanistic and experimental validation in ponatinib-associated cardiotoxicity.

## 1. Introduction

Tyrosine kinase inhibitors (TKIs) directed against BCR::ABL1 have transformed the management of Philadelphia chromosome-positive leukemias, particularly in chronic myeloid leukemia (CML) and Philadelphia chromosome-positive acute lymphoblastic leukemia (Ph-positive ALL), by suppressing oncogenic kinase signaling [1]. Nonetheless, a subset of patients experience relapse or treatment failure because ABL1 kinase-domain mutations impair inhibitor binding, most notably the T315I gatekeeper substitution, which confers broad resistance to earlier-generation TKIs [2]. Ponatinib is a third-generation BCR::ABL1 TKI designed to inhibit T315I while retaining activity against most other resistance mutations. It shows potent antileukemic efficacy in patients with relapsed or refractory BCR::ABL1-positive disease [3]. Ponatinib inhibits multiple kinases, including angiogenic receptors such as vascular endothelial growth factor receptor (VEGFR), fibroblast growth factor receptor (FGFR), and platelet-derived growth factor receptor (PDGFR), besides BCR::ABL1. This indicates a broader target spectrum than that of earlier BCR::ABL1 TKIs [4]. Clinical studies have reported increased rates of arterial occlusive events, myocardial infarction, cerebrovascular events, hypertension, and heart failure in patients treated with ponatinib, leading to label changes, dose optimization strategies, and recommendations for strict cardiovascular risk assessment and monitoring [5]. Accordingly, delineating how ponatinib’s kinase-inhibition profile contributes to cardiovascular toxicity is a major clinical priority, particularly in later-line settings such as T315I or multi-TKI-resistant/intolerant disease. Such mechanistic insight may also inform strategies to mitigate off-target toxicity.

Experimental studies in cardiomyocytes, endothelial cells, platelets, and myeloid lineages suggest that ponatinib perturbs signaling pathways involved in cardiomyocyte survival, mitochondrial function, endothelial integrity, thrombosis, and innate immune activation [6]. In cardiac models, including zebrafish hearts and neonatal rat ventricular cardiomyocytes, ponatinib suppresses pro-survival AKT and extracellular signal-regulated kinase (ERK) signaling, reduces cardiomyocyte proliferation, and induces apoptosis. These are consistent with direct injury to cardiac muscle cells [6]. Studies in human induced pluripotent stem cell-derived cardiomyocytes and in ponatinib-exposed mice have reported impaired mitochondrial adenosine triphosphate (ATP) synthesis and activation of a mitochondrial integrated stress response, implicating mitochondrial stress in ponatinib-induced contractile dysfunction [7]. Endothelial and platelet experiments indicate that ponatinib promotes endothelial apoptosis, impairs angiogenic responses, induces an inflammatory phenotype, and enhances platelet activation and arterial thrombus formation [8]. This supports a potential contribution of endothelial dysfunction and a prothrombotic state to the arterial events observed in ponatinib-treated patients [9]. Activation of an innate immune signaling axis involving S100A8/A9 and NLRP3 in cardiac and systemic myeloid cells further supports a role for innate immune activation in ponatinib cardiotoxicity [10]. Therefore, the diverse pathway perturbations observed in cardiomyocytes, endothelial cells, platelets, and myeloid lineages indicate that ponatinib cardiotoxicity is complex and multifactorial, with contributions from cardiomyocyte injury, mitochondrial dysfunction, endothelial damage, thrombosis, and innate immune activation [11].

Although previous experimental studies have documented ponatinib-mediated injury in various cardiac and vascular models, the transcriptomic basis underlying drug-specific toxicity remains unclear. Therefore, we conducted a stratified meta-analysis of heterogeneous cancer cell line transcriptomes to derive a robust ponatinib-associated transcriptional signature with potential relevance to cardiovascular toxicity. Subgroup-specific differential expression analysis was followed by fixed-effect meta-analysis to obtain pooled effect estimates across control-defined subgroups. We then assessed cross-model consistency using transcriptomic profiles from human induced pluripotent stem cell (iPSC)-derived cardiomyocytes and analyzed imatinib in parallel under an identical workflow to distinguish ponatinib-specific transcriptional responses from shared kinase-inhibitory effects.

Prior computational studies have shown that integrative network-based analyses can uncover molecular links among cardiovascular disorders and support candidate prioritization from complex omics datasets [12]. More broadly, data-driven computational approaches have been increasingly used to extract biologically and clinically meaningful information from large-scale biomedical data [13]. Guided by these developments, we conducted a stratified meta-analysis of heterogeneous cancer cell line transcriptomes and comparative analyses with an independent human iPSC-derived cardiomyocyte line to derive a robust ponatinib-associated transcriptional signature with potential implications for cardiovascular toxicity.

## 2. Results

**Figure 1:**
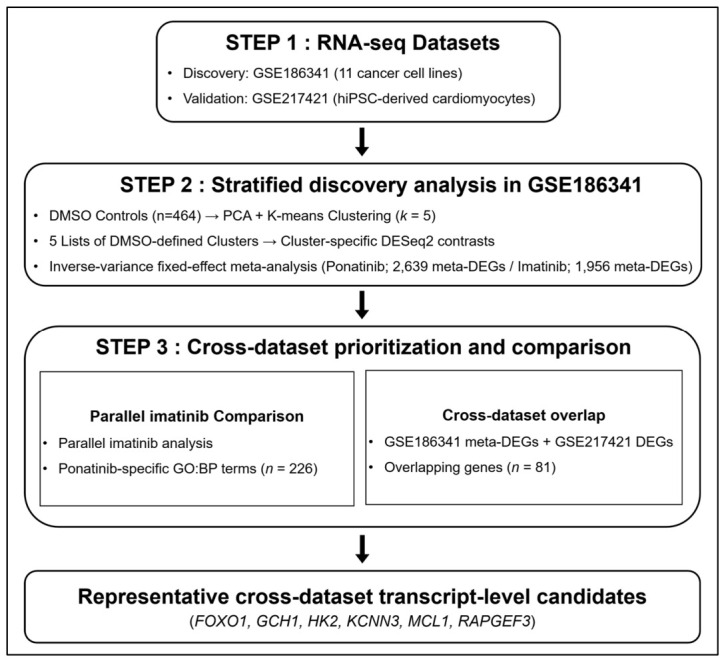
Integrative transcriptomic framework for identifying ponatinib-associated cardiotoxicity signatures. Public RNA-seq datasets were curated from the Gene Expression Omnibus (GEO), including GSE186341 for discovery and GSE217421 for external validation. In GSE186341, dimethyl sulfoxide (DMSO) control samples were stratified by principal component analysis (PCA) followed by *k*-means clustering, and ponatinib- and imatinib-associated differential expression was estimated by cluster-specific DESeq2 contrasts followed by fixed-effect meta-analysis. Cross-dataset overlap analysis with GSE217421 and parallel comparison with imatinib were then used to prioritize transcript-level candidates and ponatinib-specific Gene Ontology Biological Process (GO:BP) terms.

The first dataset (GSE186341) contained whole-transcriptome profiles from 11 cancer cell lines perturbed by 32 kinase inhibitors, dimethyl sulfoxide (DMSO), and water as controls (Figure 1) [13]. The second dataset (GSE217421) consists of RNA-seq profiles from iPSC-derived cardiomyocytes treated with 54 FDA-approved drugs, including multiple kinase inhibitors, with DMSO controls across six independent donor-derived lines [14]. In GSE186341, DMSO-treated controls (*n* = 464) outnumbered ponatinib-treated cases (*n* = 20) by over 20-fold. Given the pronounced imbalance between the groups, we examined the transcriptomic structure of the controls using principal component analysis (PCA) on the top 10% of the most variable genes [15].

**Figure 2:**
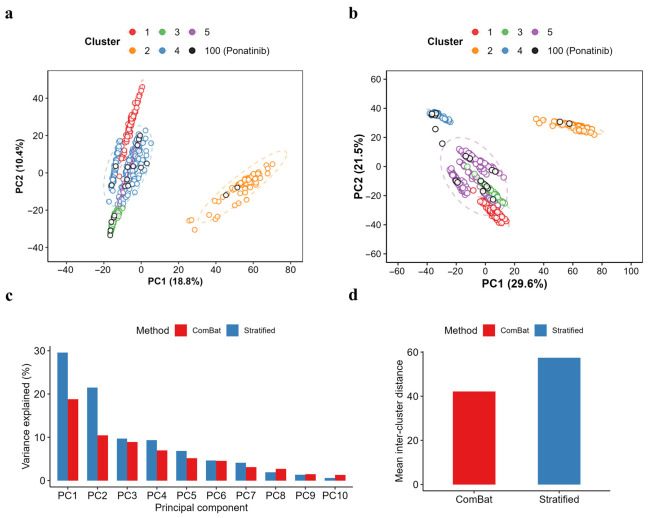
Principal component structure of DMSO-treated transcriptomes and comparison of two analysis strategies: (**a**,**b**) PCA of DMSO-treated samples is shown as PC1 versus PC2 (variance explained is indicated on each axis). Samples are colored by cluster assignment (Clusters 1–5). Ponatinib-treated samples are overlaid as black-edged points to enable direct visual comparison against the DMSO-derived cluster structure. Dashed ellipses indicate 95% cluster regions. (**c**) Variance explained by the first 10 principal components is compared between the two strategies (“ComBat” and “Stratified”). (**d**) Mean inter-cluster distance under each strategy, summarizing overall cluster separation.

PCA revealed a marked substructure within the controls, with distinct separation along principal component (PC) 1 (29.6%) and PC2 (21.5%), suggesting that hidden batch effects may exist even within a nominally unified group (Figure 2b and Figure S1). ComBat is widely recognized as a representative method for batch correction [16]. However, given the heterogeneous multi-cell-line composition of this transcriptomic dataset (GSE186341) and the possibility that a single linear batch-adjustment model may attenuate biologically meaningful variation, we partitioned the DMSO controls into five clusters by *k*-means to preserve the intrinsic heterogeneity of the control group (Figure 2b–d and Figures S1–S3). We then combined subgroup-specific treatment-effect estimates using fixed-effect meta-analysis to prioritize genes showing reproducible, directionally concordant ponatinib-associated changes across subgroup-specific contrasts after DMSO-based stratification of the controls [17].

The variance-explained curve exhibited a clear elbow around PC3–PC4, indicating sharply diminishing marginal gains. By PC20, the cumulative variance explained was 91.4%, capturing the vast majority of the transcriptomic variation, beyond which additional components contributed negligibly (Figure S4a). Accordingly, we retained the PC1–PC20 subspaces for clustering. We evaluated cluster numbers using average silhouette and within-cluster sum of squares (WCSS) [18,19]. Silhouette rose from 0.05 (*k* = 2) to 0.28 (*k* = 10) without reaching a plateau (Figure S4c), whereas the WCSS decreased smoothly with increasing k (Figure S4b), indicating that none of the metrics identified distinct optimal values. To refine the decision, we assessed clustering stability using the bootstrap Jaccard, prediction strength, and proportion of ambiguous clustering (PAC) [20,21]. Jaccard indices generally declined with increasing k and were low at *k* = 5 and *k* = 6 (0.181 at *k* = 5; 0.185 at *k* = 6). The prediction strength approached the conventional 0.80 threshold (0.768 at k = 5; peak 0.897 at *k* = 6). PAC indicated modest ambiguity at *k* = 5 (0.091) but near-zero ambiguity at *k* = 6 (0.000) (Table S1). Although *k* = 6 showed slightly stronger stability indices, the gain over *k* = 5 was small, whereas *k* ≥ 9 produced much smaller clusters (minimum *n* = 22–28; Table S2). Considering both stability and cluster-size suitability for downstream subgroup comparison, *k* = 5 was selected as the final solution, yielding cluster sizes of *n* = 60–212 (median = 60).

**Figure 3:**
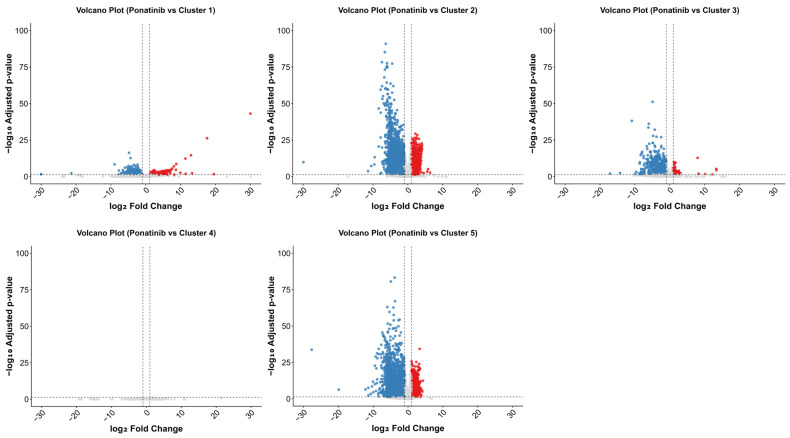
Differential expression of ponatinib-treated samples relative to heterogeneous DMSO control clusters. Volcano plots show differential gene expression in ponatinib-treated samples compared with each of the five DMSO-defined control clusters (Clusters 1–5; defined in Figure 2), reflecting distinct baseline transcriptional states. Each panel displays log_2_ fold change (x-axis) versus −log_10_ adjusted *p*-value (y-axis). Red and blue points indicate significantly upregulated and downregulated genes, respectively (false discovery rate (FDR)-adjusted *p* < 0.05). Vertical dashed lines denote the log_2_ fold-change threshold, and the horizontal dashed line denotes the significance threshold.

The five subgroups were derived exclusively from the DMSO-treated samples and served as matched comparators for the ponatinib-treated samples. We generated subgroup-specific lists of differentially expressed genes and combined them to define a consolidated ponatinib-associated transcriptomic signature. Ponatinib-treated samples were compared with each of the five DMSO-derived control clusters, which revealed substantial differences in the extent of differential gene expression across the clusters (Figure 3; see also Figure S5 for imatinib), suggesting that the ponatinib response was influenced by the transcriptional background of the reference control cluster. In agreement with this interpretation, at a false discovery rate (FDR) < 0.05, the number of significantly differentially expressed genes (DEGs) was as follows: cluster 1, *n* = 673; cluster 2, *n* = 6962; cluster 3, *n* = 2126; cluster 4, *n* = 0; and cluster 5, *n* = 6855. Applying the same framework to imatinib revealed comparable subgroup-dependent variability: Cluster 1, *n* = 27; Cluster 2, *n* = 6853; Cluster 3, *n* = 1221; Cluster 4, *n* = 7480; Cluster 5, *n* = 5. Thus, consistent with ponatinib, imatinib showed pronounced cluster-dependent variability.

**Figure 4:**
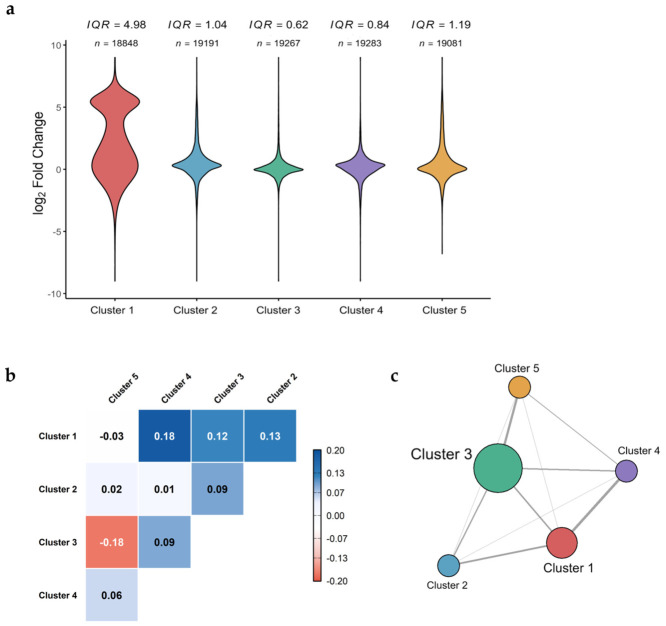
Distribution and concordance of ponatinib-associated fold-change profiles across DMSO-defined control clusters: (**a**) Violin plots summarize the distribution of gene-wise log_2_ fold changes for ponatinib versus each DMSO-defined cluster (Clusters 1–5). The interquartile range (IQR) and the number of genes included (n) are annotated above each cluster. (**b**) Heatmap of pairwise Pearson correlations (*r*) between cluster-specific log_2_ fold-change profiles, showing overall low concordance across clusters (*r* range shown in the panel). (**c**) Network representation of the correlation matrix, where nodes represent clusters and edges reflect pairwise correlations; node size is proportional to the mean correlation of each cluster with all others.

Pearson correlations of fold-change estimates further showed limited concordance across clusters in ponatinib-treated samples (Figure 4b–c), underscoring the importance of control stratification in capturing reproducible drug-associated signatures in heterogeneous transcriptomic datasets. Among the five clusters, Cluster 1 and Cluster 4 showed the highest correlation, yet this remained statistically weak (*r* = 0.18, 95% CI [0.17–0.20]), while the dispersion of log_2_ fold-change estimates varied substantially across clusters (Figure 4a–b). Similarly, pairwise correlations across all clusters were low (−0.18 to 0.18; mean Pearson’s *r* = 0.05), indicating that fold-change estimates were highly dependent on the reference cluster used (e.g., Cluster 2 vs. Cluster 3, *r* = 0.009). Cluster 3 was clearly separated as the central node of divergence within the correlation-based network (Figure 4c) and exhibited minimal correlation with the other clusters.

**Figure 5:**
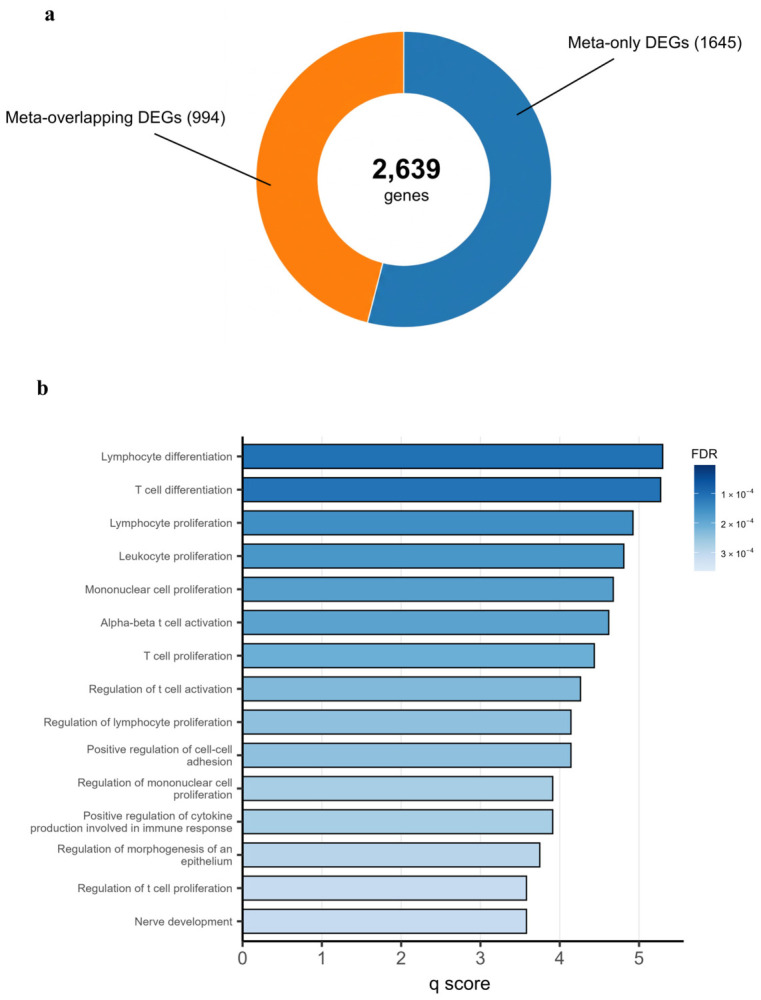
Composition of meta-DEGs and Gene Ontology enrichment of downregulated signatures following ponatinib treatment: (**a**) Donut chart summarizing meta-DEGs identified by meta-analysis across the five DMSO-defined control clusters. Meta-only DEGs are significant only in the meta-analysis, whereas meta-overlapping DEGs are significant in the meta-analysis and in at least one cluster-specific comparison. Counts for each category are indicated. (**b**) Bar plot of Gene Ontology (GO) Biological Process enrichment for downregulated meta-DEGs (log_2_FC < −1.0, false discovery rate (FDR) < 0.05). The x-axis shows the q score (as displayed), and the color scale indicates adjusted *p*-values.

These observations suggested substantial heterogeneity among the control subgroups, with only 19 genes common to all five clusters (Figure 5a and Figure S6; see also Figure S7 for imatinib). All 19 genes showed concordant downregulation across the five cluster-specific comparisons (Table S3). *CALM1*, *HSPA9*, and *WNT7B* suggest possible links of the 19-gene overlap to vascular injury, mitochondrial stress, and Wnt/β–catenin signaling perturbation. *CALM1* has been linked to calcium-dependent endothelial homeostasis and angiogenesis [22], *HSPA9* to mitochondrial protein folding and stress adaptation [23], and *WNT7B* to Wnt/β–catenin signaling and tissue remodeling [24,25]. However, marked subgroup heterogeneity limited direct cluster overlap to a narrow view of ponatinib-associated transcriptional alteration, despite previous reports describing ponatinib as affecting multiple biological and transcriptional axes [6,7,8,9,10].

Although batch correction can reduce technical variation, it may also suppress biologically relevant variations across heterogeneous DMSO controls [26]. Rather than eliminating this variability, we conducted a meta-analysis of cluster-specific transcriptomic responses to ponatinib in GSE186341. The pooled analysis identified 2639 ponatinib-associated meta-DEGs, of which 1645 were uniquely detected by meta-analysis, indicating the benefit of integrative comparison across the five distinct DMSO control clusters (Figure 5a). Functional enrichment of the 2639 meta-DEGs revealed broad immune-related signatures, including both proinflammatory and regulatory pathways (Figure S8). Given the transcriptional dysregulation observed in (or characteristic of) the TKI-treated group (Figure S9), we subsequently focused on ponatinib-downregulated meta-DEGs to delineate the selective repression of innate immune pathways.

A broader convergent ponatinib-associated signature comprising 2639 meta-DEGs was recovered by meta-analysis across the heterogeneous control clusters, with lymphocyte and T-cell differentiation and activation, cytokine production, and cell–cell adhesion emerging as leading pathways among ponatinib-downregulated meta-DEGs in GSE186341 (Figure 5b). Lymphocyte and T-cell differentiation and activation are linked to adaptive immune regulation, whereas cytokine production and cell–cell adhesion are associated with inflammatory signaling and leukocyte–endothelial interaction. Prior ponatinib studies have also described inhibition of induced programmed death-ligand 1 (PD-L1) expression, altered CD8+ T-cell activity, modulated T helper 1/T helper 2 (Th1/Th2) balance, endothelial inflammation, and procoagulant change. T-cell activation and proinflammatory cytokine signaling are also implicated in cardiac injury and remodeling in cardiovascular disease [27,28].

**Figure 6:**
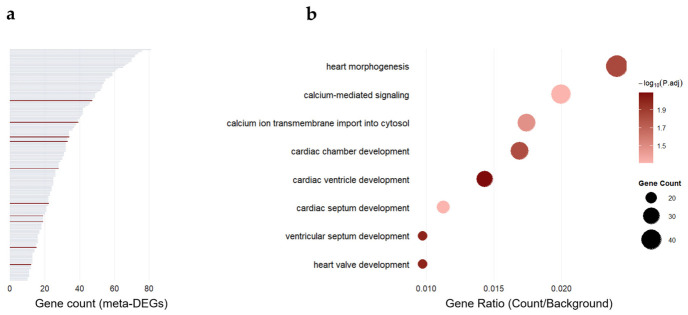
Gene Ontology Biological Process enrichment of ponatinib-associated meta-DEGs after excluding imatinib-shared terms: (**a**) Bar plot summarizing GO enrichment results for 2639 ponatinib-associated meta-DEGs after filtering out GO terms also enriched in the imatinib analysis (false discovery rate (FDR) < 0.05), yielding 226 ponatinib-specific GO terms. Bar length indicates the number of genes contributing to each term. (**b**) Bubble plot highlighting selected heart- and calcium signaling-related GO terms from the filtered ponatinib-specific enrichment set. The x-axis indicates gene ratio (Count/Background), bubble size indicates gene count, and color indicates −log_10_ (adjusted *p*-value), as shown in the legend.

We then applied the same analysis to imatinib-treated samples to determine whether the immune, vascular–inflammatory, and mitochondrial features observed in ponatinib-treated samples were also present in imatinib-treated samples. Imatinib-associated meta-DEGs were enriched in pathways not directly related to cardiovascular biology, such as cilium movement, sperm motility, and retinoid metabolism (Figures S9 and S10). These imatinib-enriched terms (FDR < 0.05, gene count ≥ 10) were subsequently excluded from the ponatinib enrichment profile, yielding 226 processes defined as ponatinib-specific (Figure 6a and Figure S10a). Among these, eight terms were related to cardiac morphogenesis, myocardial contractility, calcium handling, or vascular structural processes closely linked to cardiotoxic responses [29]. None were enriched under imatinib treatment (FDR > 0.2), underscoring their specificity to ponatinib-induced transcriptional disruption. To highlight this divergence, ponatinib-specific cardiovascular terms are presented separately (Figure 6b and Figure S10b).

**Figure 7:**
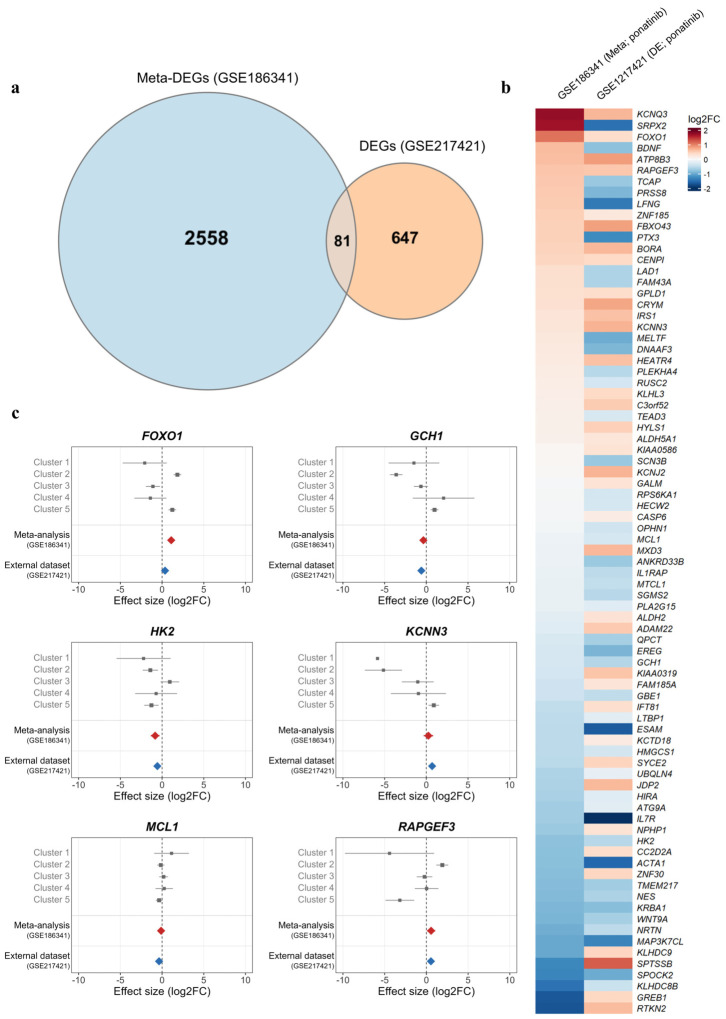
Cross-dataset overlap of the ponatinib-associated meta-signature and functional annotation of downregulated genes: (**a**) Venn diagram showing the overlap between ponatinib-associated meta-differentially expressed genes (DEGs) from GSE186341 (meta-analysis) and DEGs from the independent dataset GSE217421, yielding 81 shared genes. (**b**) Heatmap of log_2_ fold-change values for the 81 shared genes in each dataset (GSE186341 meta-analysis and GSE217421 differential expression), visualizing the direction and magnitude of changes across datasets. (**c**) Forest plots showing cluster-specific log_2_ fold-change estimates in GSE186341 (Clusters 1–5), the pooled fixed-effect meta-analytic estimates, and differential-expression estimates from GSE217421 for six selected genes (*FOXO1*, *GCH1*, *HK2*, *KCNN3*, *MCL1*, and *RAPGEF3*). Squares indicate effect estimates for individual clusters, with horizontal lines indicating 95% confidence intervals. Diamonds denote the pooled meta-analytic estimate and the estimate from GSE217421, allowing direct comparison of effect direction and magnitude across the discovery and external datasets.

We next analyzed GSE217421, an independent dataset of iPSC-derived cardiomyocytes, to examine the reproducibility of ponatinib-associated transcriptional alterations in a cardiac model [14]. Owing to its single-batch design, this dataset was not applicable to batch correction or meta-analytic integration and was therefore analyzed independently. The cross-dataset intersection between 2639 ponatinib-associated meta-DEGs (GSE186341) and DEGs from GSE217421 yielded 81 consistently dysregulated genes (Figure 7a). Among the 81 overlapping genes, 49 showed concordant direction of expression change between the two datasets (Figure 7b and Table S5). We adapted the cluster-based meta-analysis to imatinib treatment, using the same framework as that applied to ponatinib, and identified 1950 meta-DEGs in GSE186341 to assess whether the observed transcriptional responses were specific to ponatinib. A separate DEG analysis of GSE217421 under imatinib treatment refined the 1950 meta-DEGs to 4 genes (Figure S11), yielding far fewer meta-DEGs than ponatinib (4 vs. 81). None of the 81 ponatinib-specific genes overlapped with the 4 imatinib-specific DEGs, indicating a drug-specific transcriptional response.

The six genes (*KCNN3*, *RAPGEF3*, *FOXO1*, *HK2*, *MCL1*, and *GCH1*) showed ponatinib-exclusive directional consistency between the pooled meta-analytic estimates and the external cardiomyocyte dataset (Figure 7c and Table S6), supporting transcriptional alterations related to ion-channel homeostasis, insulin-signaling balance, cAMP-responsive stress signaling, and mitochondrial redox control, all of which have been linked to ponatinib-associated cardiotoxic phenotypes [29]. Functionally, *KCNN3* and *RAPGEF3* contribute to cardiac repolarization-associated ion handling and cAMP-mediated cardiac stress signaling, respectively; dysregulation of both genes may increase susceptibility to electrophysiological instability and stress-responsive remodeling [30,31,32]. Both *FOXO1* and *HK2* regulate insulin-responsive metabolism and glucose utilization in cardiomyocytes, and dysregulation of both genes implies metabolic stress and impaired energetic adaptation [33,34,35]. Finally, *MCL1* and *GCH1* contribute to mitochondrial homeostasis and redox regulation, respectively; dysregulation of both genes may aggravate oxidative injury and compromise cardiomyocyte survival [36,37,38].

## 3. Discussion

In this study, we conducted a subgroup-stratified meta-analysis of cancer cell line transcriptomes profiled under multiple kinase inhibitor treatments and derived a robust ponatinib-associated transcriptional signature with potential relevance to cardiovascular toxicity. A total of 81 genes in the ponatinib-associated signature were also observed in an independent human iPSC-derived cardiomyocyte dataset (Figure 7a,b). Prior experimental studies have reported ponatinib-associated cardiovascular toxicity involving stress response programs and inflammatory signaling [7], including the integrated stress response and S100A8/A9–NLRP3–IL-1β-linked innate immune activation [10], together with attenuation of cardioprotective AKT/ERK signaling [6]. Recent cardiovascular literature has discussed oxidative stress modulation within a broader framework of cardiovascular injury, inflammatory remodeling, and repair [39,40]. The ponatinib-associated oxidative stress signals identified here are therefore interpreted as transcript-level candidates for subsequent mechanistic evaluation rather than as direct evidence of established repair mechanisms.

Pathway enrichment analysis for the 81 transcripts shared between datasets did not yield any term that survived FDR correction. A negative FDR result does not by itself exclude biologically coherent information, because enrichment significance is highly sensitive to database composition, category size and granularity, overlap among annotation terms, background selection, sample size, and multiple-testing correction [41,42,43,44,45,46,47]. Nominal *p*-values were therefore not used as an independent basis for mechanistic claims. Functional interpretation was instead based on gene-level annotation of concordantly dysregulated transcripts together with prior ponatinib literature describing mitochondrial dysfunction with integrated stress response activation, inflammatory injury, and AKT/ERK suppression [6,7].

GSE186341 was not used to infer cardiomyocyte biology directly. Instead, it was used at the discovery stage to identify ponatinib-associated transcript-level candidates recurring across heterogeneous cellular backgrounds. A major challenge in the discovery dataset was marked baseline heterogeneity across cancer cell lines, which complicated unstratified differential expression analysis (Figure 2 and Figure 4b). Large perturbational transcriptomic resources have shown that drug-induced expression responses are shaped not only by drug action but also by lineage and baseline cellular state. In high-dimensional, heterogeneous transcriptomic datasets, applying linear batch correction can attenuate biological signals when the technical structure is entangled with cell line identity or the baseline cellular state [48,49]. We therefore used a stratified approach to compare ponatinib-treated samples against more locally homogeneous reference groups rather than a single pooled control set. Samples were grouped using *k*-means clustering, differential expression was then estimated within each cluster, and cluster-level effects were combined using fixed-effect meta-analysis (Figure 2, Figure 3 and Figure S4). Fixed-effect integration summarized concordant transcript-level signals across the observed DMSO-defined subgroups after control stratification. The pooled estimate supported transcript-level candidate selection within GSE186341 and was not interpreted as evidence of identical lineage-related effects across all cancer cell lines. Nevertheless, the DMSO-defined clusters may still reflect cell-line identity, tissue origin, and baseline cellular state [50,51], and the resulting meta-significant genes should therefore be interpreted as conservative cross-subgroup candidates rather than as evidence of identical biology across all cancer cell lines.

The study did not include new in vitro or in vivo validation experiments. The analytical strategy therefore aimed to prioritize a more reproducible set of transcript-level candidates rather than to establish mechanisms. Subgroup-level analysis in GSE186341, fixed-effect integration across control-defined clusters, and external transcript-level evaluation in an independent human iPSC-derived cardiomyocyte dataset were used to reduce false-positive candidate selection and improve reproducibility. Despite the analytical steps taken to reduce false-positive candidate selection and improve reproducibility, several limitations remained: (**1**) Genome-wide transcriptomic analysis does not directly establish protein quantity, protein function, ion-channel activity, mitochondrial function, or cardiomyocyte contractile function [52,53]. (**2**) Differences in cell type, drug exposure duration, drug concentration, and sequencing conditions across datasets also complicate direct comparison across studies and limit mechanistic interpretation and subsequent preclinical investigation [50,51]. (**3**) Human iPSC-derived cardiomyocytes show structural and electrophysiological immaturity, inter-line variation, and markedly lower *KCNJ2* expression than adult human left ventricular tissue, which limits extrapolation to adult human cardiomyocyte biology [54,55,56,57].

Ponatinib-associated signature genes identified here represent transcriptome-level candidates of ponatinib-associated cardiovascular toxicity and may guide subsequent mechanistic studies and experimental validation [14,58,59]. In conclusion, the integrated analysis narrowed the list of ponatinib-associated transcript-level candidates by identifying a set of genes supported across the discovery dataset and an independent human iPSC-derived cardiomyocyte dataset. The prioritized candidates may provide a focused basis for subsequent mechanistic and experimental validation in ponatinib-associated cardiovascular toxicity.

## 4. Materials and Methods

### 4.1. Data Acquisition and Preprocessing

No physical instruments or wet-laboratory reagents were used in this study because all analyses were based entirely on publicly available RNA-seq datasets. Public RNA-seq datasets were obtained from the Gene Expression Omnibus (GEO) database using the following inclusion criteria: human bulk RNA-seq, TKI exposure with DMSO controls, availability of raw gene-level counts, and at least two biological replicates per condition [60]. Two datasets met these criteria. The GSE186341 dataset includes transcriptome-wide RNA-seq profiles from 11 human cancer cell lines treated with 32 kinase inhibitors and DMSO controls (*n* = 464). Drug annotations were harmonized with compound-level identifiers, and only samples treated with ponatinib (*n* = 20) or imatinib (*n* = 20) were retained for downstream analyses. GSE217421 provides RNA-seq profiles of human iPSC-derived cardiomyocytes generated from six independent donor lines. This dataset included 22 ponatinib-treated samples, 63 matched DMSO controls, and 21 imatinib-treated samples. Within each dataset, raw gene-count tables were assembled into a unified gene-by-sample count matrix for analysis [61]. Normalization was performed in DESeq2 (v1.38.3) using size-factor estimation to account for library depth differences. For exploratory analyses (PCA and clustering), normalized counts were log-transformed to log_2_ (count + 1), whereas differential expression (DE) testing was conducted on the raw counts within the DESeq2 modeling framework. Gene identifiers were mapped and annotated using the org.Hs.eg.db package (v3.15.0) [62].

### 4.2. Control Stratification and Clustering

PCA was performed on DMSO-only samples using the top 10% of the most variable genes, defined by the variance of log-transformed expression values, to characterize the heterogeneity within the DMSO controls of GSE186341. The first 20 principal components (PC1–PC20) were retained to represent the dominant transcriptomic structure while avoiding redundant higher-order components, guided by scree-profile drop-off (Figure S4). K-means clustering was then applied to the PC1–PC20 space to partition the DMSO controls into transcriptionally coherent subgroups. Cluster number (*k*) was evaluated using the within-cluster sum of squares, average silhouette width, and stability metrics, including bootstrap Jaccard indices (*B* = 200), prediction strength (two-fold resampling; 100 repetitions), and the PAC from consensus clustering (80% subsampling; 200 iterations) [21,63]. Candidate solutions producing clusters with fewer than 15 samples were treated as non-representative and excluded from the final selection. Based on the combined stability–representativeness tradeoff, *k* = 5 was selected. Ponatinib-treated samples were projected onto the same PCA embedding for visualization but were not used to fit or define the DMSO-derived clusters. A global batch-adjustment approach (e.g., ComBat) was considered but not applied to avoid attenuating structured biological differences that track the baseline cellular state in a multi-cell-line panel [16]. Instead, downstream inference was conducted by comparing each treatment group against the stratified DMSO subgroups using cluster-wise contrasts.

### 4.3. Differential Expression and Meta-Analysis

Cluster-wise DE analysis was performed in DESeq2 using the design formula: PC1 + PC2 + PC3 + treatment [61]. PC1–PC3 were included as covariates to account for residual within-cluster structure not captured by cluster assignment alone. Statistical significance was defined as FDR < 0.05, without imposing an additional absolute log_2_ fold-change threshold at the DE testing step. For each gene, cluster-specific effect estimates (log_2_ fold-changes) and standard errors were integrated using an inverse-variance-weighted fixed-effect meta-analysis implemented in the metafor package (v4.2-0) [64]. Genes were retained if they satisfied the following criteria: (**1**) available data in at least two contrasts, (**2**) directional consistency in ≥ 4 clusters, (**3**) low heterogeneity (I^2^ < 50%), and (**4**) two-sided meta-analysis *p* < 0.05. Genes passing these criteria but absent from any single-cluster DEG list were labeled “meta-only DEGs.”

### 4.4. Connectivity and Comparative Analysis

To quantify the transcriptomic connectivity across cluster-wise responses, pairwise Pearson correlations were computed between genome-wide log_2_ fold-change vectors obtained from each cluster-specific contrast. The resulting correlation structure was represented as a weighted network in iGraph R package (https://igraph.org/, accessed on 29 April 2026), with edge weights defined by absolute correlation magnitude and node size scaled by betweenness centrality [65]. An identical stratification-DE-meta-analysis workflow was applied to imatinib-treated samples in GSE186341 and imatinib-treated cardiomyocytes in GSE217421 to enable drug-to-drug comparison under matched analytic assumptions. Genes meeting the ponatinib criteria but not the corresponding imatinib criteria were classified as ponatinib-specific. External validation of GSE217421 was performed by DE analysis of ponatinib-treated versus control cardiomyocytes using a standard design (treatment). Cross-dataset reproducibility was summarized by set overlap (Venn diagrams) and Pearson’s correlation of log_2_ fold-change estimates between the discovery meta-analysis (GSE186341) and validation analysis (GSE217421).

### 4.5. Functional Enrichment and Visualization

Over-representation analysis (ORA) for Gene Ontology Biological Process (GO:BP) terms was performed in clusterProfiler (v4.6.0), separately for upregulated and downregulated gene sets [66]. Ponatinib-specific GO:BP terms were defined as terms significantly enriched under ponatinib treatment but not under imatinib treatment, using FDR < 0.05 and gene count ≥ 10 as filtering criteria [67]. All analyses were conducted in R (v4.2.2; R Foundation for Statistical Computing, Vienna, Austria). The primary R packages used were DESeq2, metafor, clusterProfiler, ComplexHeatmap, eulerr, ggplot2, and igraph. Random seeds were fixed for clustering, resampling, and consensus to ensure analytical reproducibility.

## Data Availability

All data analyzed in this study are publicly available from the NCBI Gene Expression Omnibus (GEO) under accession numbers GSE186341 and GSE217421. Analysis scripts are available from the corresponding author upon reasonable request.

## Supplementary Materials

The following supporting information can be downloaded at: https://www.mdpi.com/article/10.3390/ijms27094058/s1.
