# Supplementary material for: A Ponatinib-Associated Transcriptomic Signature: Implications for Cardiovascular Toxicity
Source: Int J Mol Sci. 2026 Apr 30;27(9):4058. doi: 10.3390/ijms27094058 (PMC13164345; doi:10.3390/ijms27094058)
Supplement: Supplementary file 1 [file ijms-27-04058-s001.zip › ijms-4190959-supplementary.pdf]

A Ponatinib-Associated Transcriptomic Signature: Implications for Cardiovascular Toxicity

Joonho Kong[1†], Jaeyeon Jang[2†], Jee Hyun Kong[2*], Taesic Lee[3*]

Department of Medicine, Yonsei University Wonju College of Medicine, 20 Ilsan-ro, Wonju 26426, Republic of Korea
Division of Hematology-Oncology, Department of Internal Medicine, Yonsei University Wonju College of Medicine, 20 Ilsan-ro, Wonju 26426, Republic of Korea
Department of Convergence Medicine, Yonsei University Wonju College of Medicine, 20 Ilsan-ro, Wonju 26426, Republic of Korea

† These authors contributed equally and share first authorship.

* Correspondence: J.H.K. (kkongg@yonsei.ac.kr); T.L. (ddasic123@yonsei.ac.kr)

Index for the Supplementary Materials

Supplementary Figures 3

Figure S1. Comparison of PCA-based control stratification and ComBat batch correction for imatinib analyses in GSE186341. 3

Figure S2. Comparison of stratified preprocessing and ComBat batch correction for ponatinib analyses in GSE186341. 5

Figure S3. Comparison of stratified preprocessing and ComBat batch correction for imatinib analyses in GSE186341. 7

Figure S4. Scree, elbow, and silhouette analyses for DMSO control clustering in GSE186341. 9

Figure S5. Cluster-specific differential expression of imatinib-treated samples in GSE186341. 11

Figure S6. Intersections of cluster-wise DEGs in response to Ponatinib in GSE186341. 12

Figure S7. Intersections of cluster-wise DEGs in response to Imatinib in GSE186341. 13

Figure S8. Direction-agnostic GO enrichment of Ponatinib-associated meta-significant genes in GSE186341. 14

Figure S9. Meta-significant gene composition and enriched biological processes in response to Imatinib in GSE186341. 15

Figure S10. Comparative GSEA of GO terms between ponatinib and imatinib in GSE186341. 16

Figure S11. Cross-dataset overlap of the imatinib-associated gene signature between GSE186341 and GSE217421. 17

Supplementary Tables 18

Table S1. Metrics supporting the selection of k for k-means clustering of DMSO controls in GSE186341. 18

Table S2. Cluster size distributions across k-means solutions for PCA-derived subgroups of GSE186341 DMSO controls. 19

Table S3. Nineteen genes shared across all five DMSO-defined subgroups in GSE186341. 20

Table S4. Summary of differential expression and meta-analysis results for Ponatinib and Imatinib. 22

Table S5. Cross-dataset comparison of 81 overlapping ponatinib-associated genes between GSE186341 and GSE217421. 23

Table S6. Cluster-specific and cross-dataset $\log_2$ fold-change estimates for six representative ponatinib-associated genes. 25

Table S7. Summary of statistical criteria used for transcript-level candidate selection. 26

Supplementary Figures

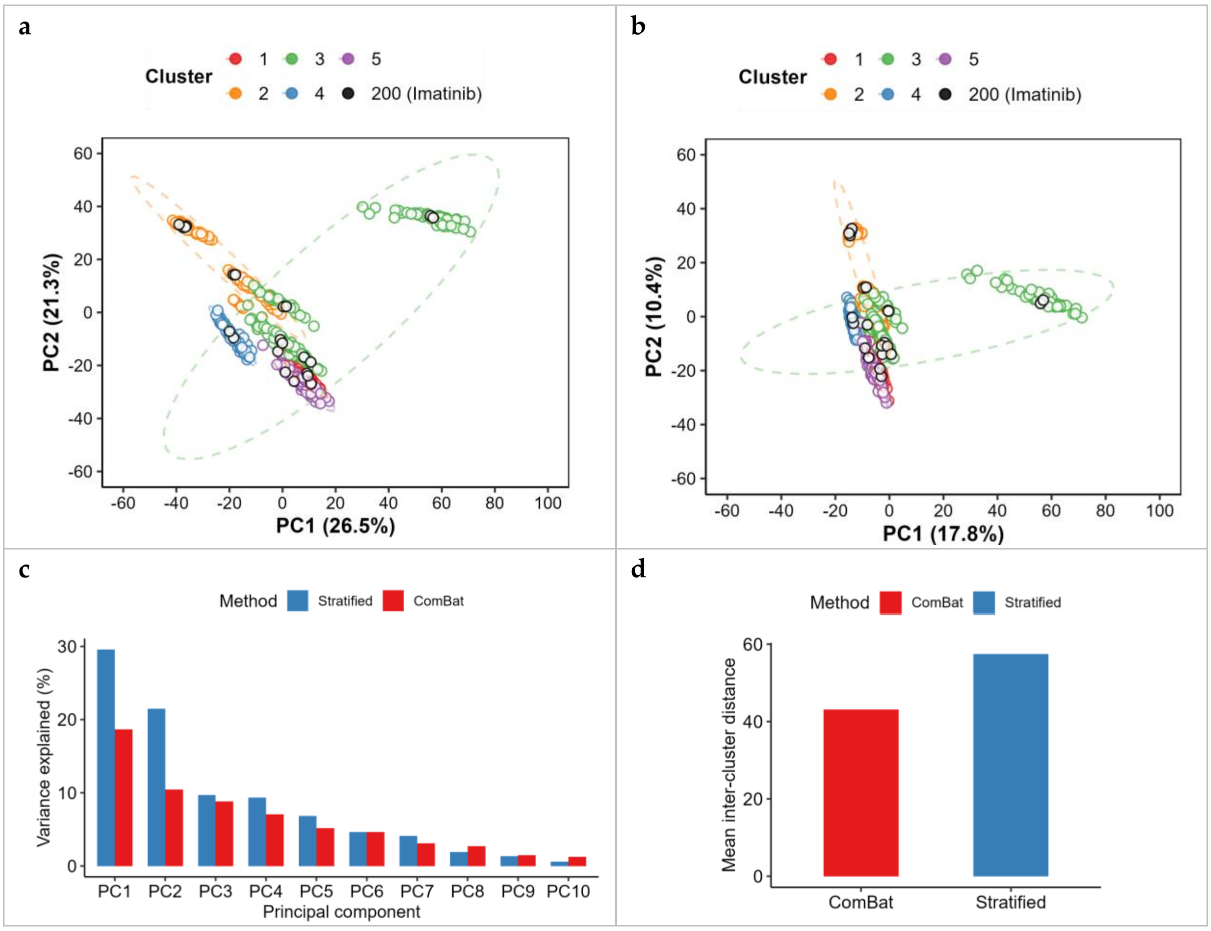

Figure S1. Comparison of PCA-based control stratification and ComBat batch correction for imatinib analyses in GSE186341.

(a) PCA of DMSO-treated samples is shown as PC1 versus PC2 (variance explained is indicated on each axis). Samples are colored by cluster assignment (Clusters 1–5) obtained from k-means clustering on the DMSO controls. Imatinib-treated samples are overlaid as black-edged points. Dashed ellipses indicate 95% cluster regions. (b) PCA of DMSO-treated samples after ComBat batch correction, shown for comparison with the stratified approach. Imatinib-treated samples are overlaid as black-edged points. Dashed ellipses indicate 95% cluster regions. (c) Variance explained by the first 10 principal components (PC1–PC10) for the two approaches (“Stratified” and “ComBat”). (d) Mean inter-cluster distance in the PC1–PC2 space for the two approaches.

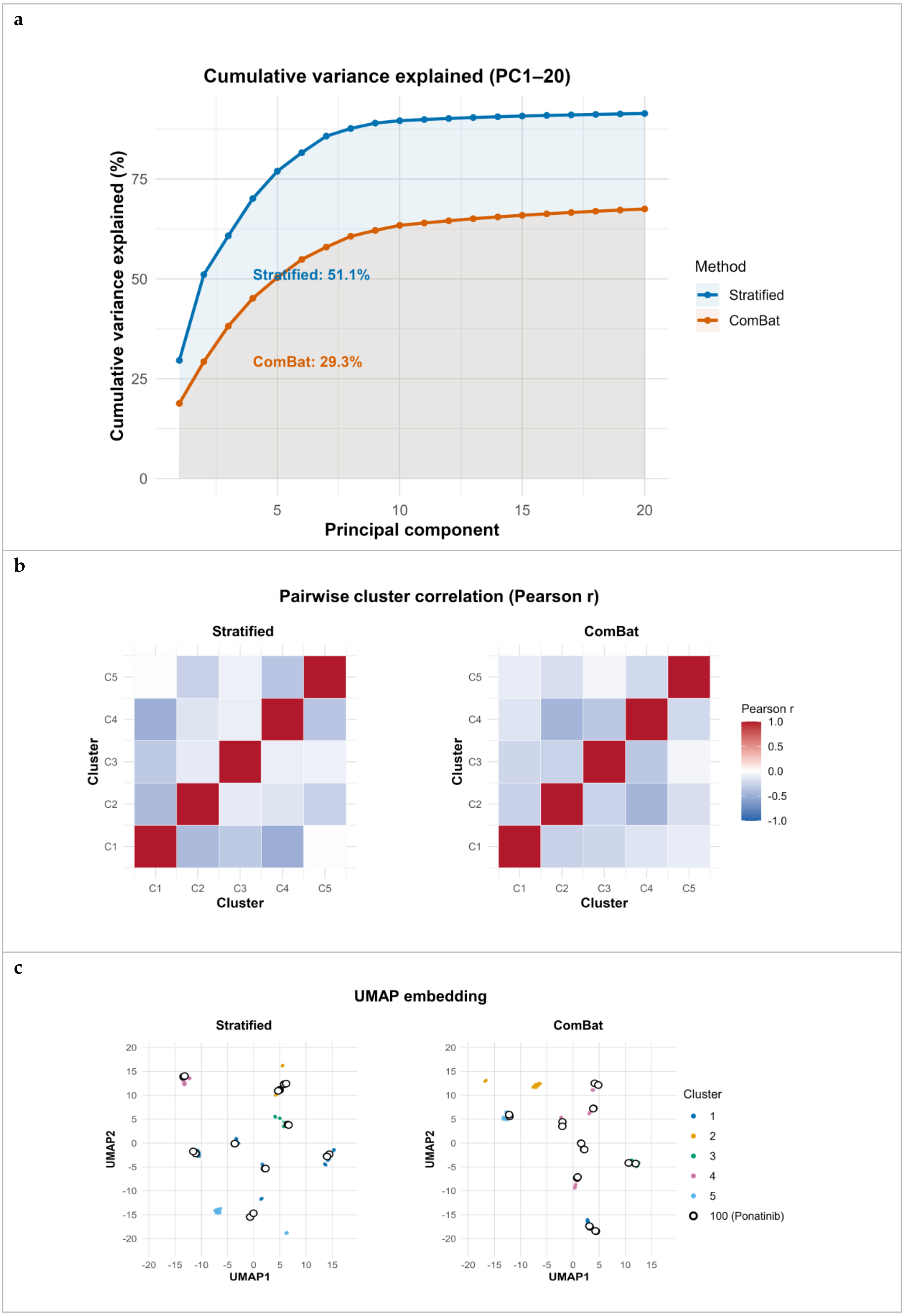

a
Cumulative variance explained (PC1–20)
Cumulative variance explained (%)
Principal component
Stratified: 51.1%
ComBat: 29.3%
Method
Stratified
ComBat
b
Pairwise cluster correlation (Pearson r)
Stratified
ComBat
Cluster
Pearson r
c
UMAP embedding
Stratified
ComBat
UMAP1
UMAP2
Cluster
100 (Ponatinib)

Figure S2. Comparison of stratified preprocessing and ComBat batch correction for ponatinib analyses in GSE186341.

(a) Cumulative variance explained by the first 20 principal components (PC1–PC20) under the two approaches ("Stratified" and "ComBat"), with summary values annotated in the panel. (b) Heatmaps of pairwise Pearson correlation coefficients (*r*) among DMSO-defined control clusters, shown separately for the stratified and ComBat approaches. (c) UMAP (Uniform Manifold Approximation and Projection) embeddings of the same samples under each approach. Points are colored by cluster assignment, and ponatinib-treated samples are indicated as black-edged points, as shown in the legend.

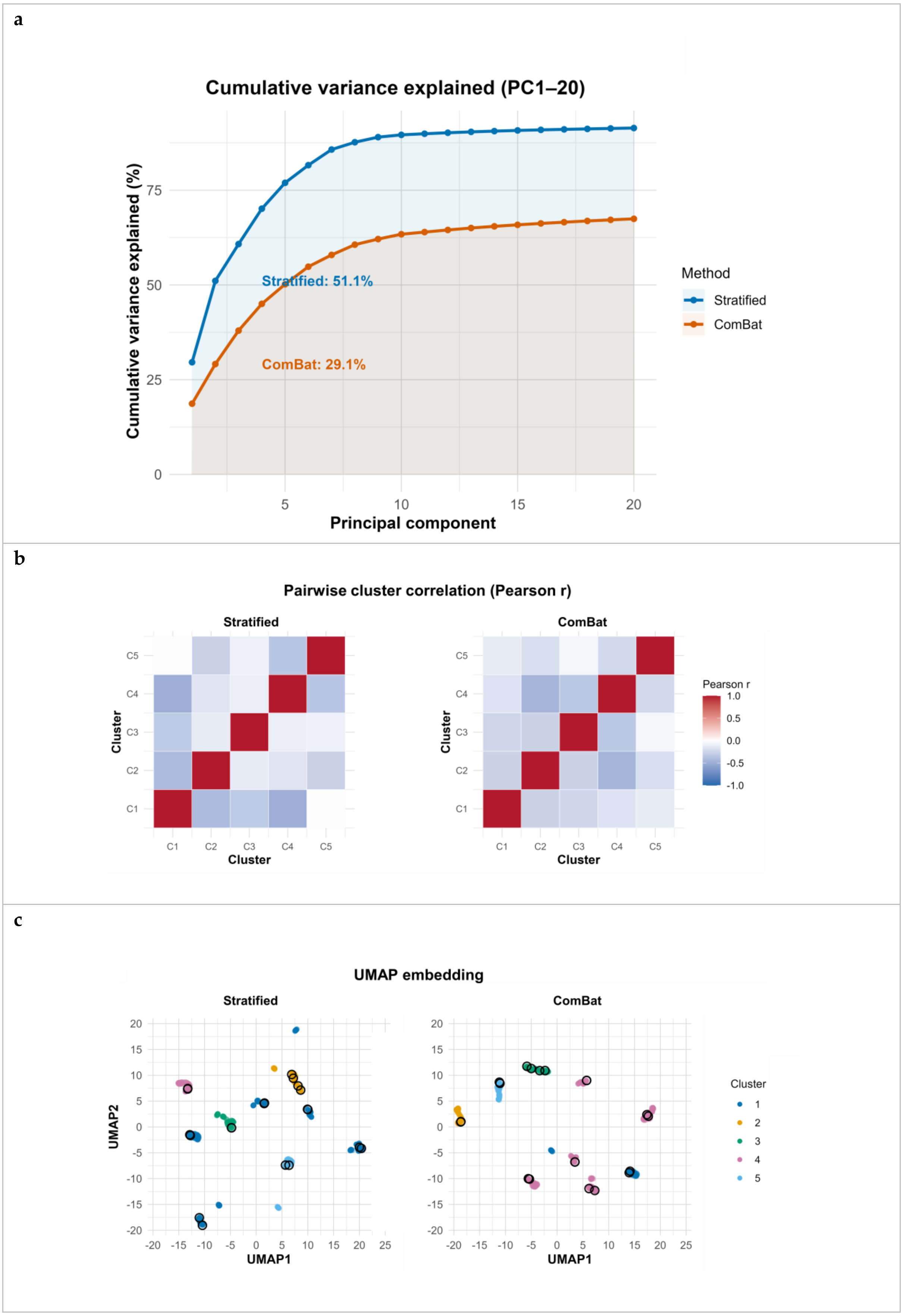
a
Cumulative variance explained (PC1–20)
Cumulative variance explained (%)
Principal component
Stratified: 51.1%
ComBat: 29.1%
Method
Stratified
ComBat
b
Pairwise cluster correlation (Pearson r)
Stratified
ComBat
Cluster
Pearson r
c
UMAP embedding
Stratified
ComBat
UMAP1
UMAP2
Cluster

Figure S3. Comparison of stratified preprocessing and ComBat batch correction for imatinib analyses in GSE186341.

(a) Cumulative variance explained by the first 20 principal components (PC1–PC20) under the two approaches ("Stratified" and "ComBat"), with summary values annotated in the panel. (b) Heatmaps of pairwise Pearson correlation coefficients (*r*) among DMSO-defined control clusters, shown separately for the stratified and ComBat approaches. (c) UMAP embeddings of the same samples under each approach. Points are colored by cluster assignment, and imatinib-treated samples are indicated as black-edged points, as shown in the legend.

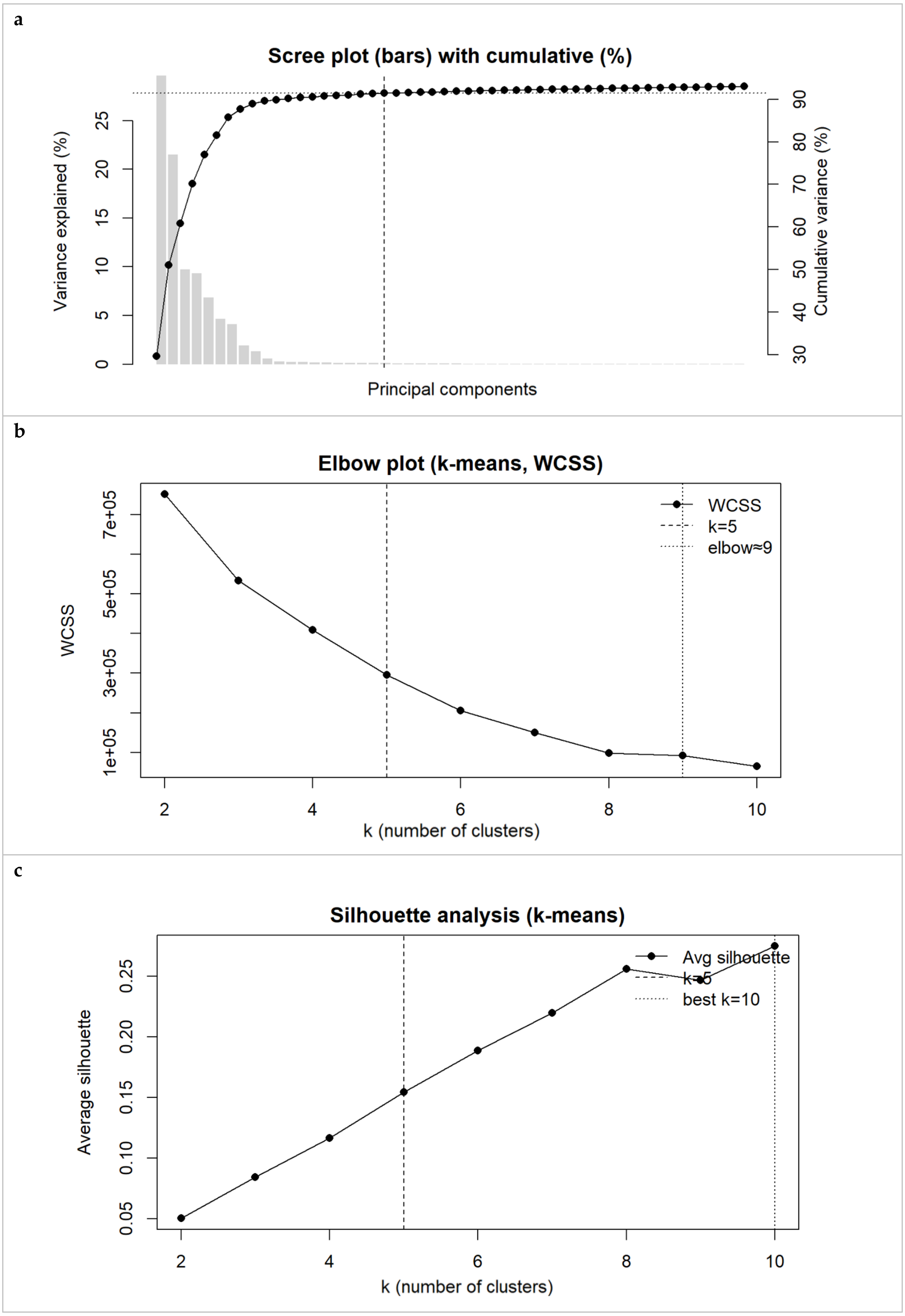
a
Scree plot (bars) with cumulative (%)
Variance explained (%)
0
5
10
15
20
25
Cumulative variance (%)
30
40
50
60
70
80
90
Principal components
b
Elbow plot (k-means, WCSS)
WCSS
1e+05
3e+05
5e+05
7e+05
WCSS
k=5
elbow≈9
2
4
6
8
10
k (number of clusters)
c
Silhouette analysis (k-means)
Average silhouette
0.05
0.10
0.15
0.20
0.25
Avg silhouette
k=5
best k=10
2
4
6
8
10
k (number of clusters)

Figure S4. Scree, elbow, and silhouette analyses for DMSO control clustering in GSE186341.

(a) Scree plot for PCA of DMSO-treated samples using the top 10% most variable genes. Bars indicate variance explained by each principal component and the line indicates cumulative variance explained (as shown in the panel). (b) Elbow plot of within-cluster sum of squares (WCSS) for k-means clustering across k = 2–10. The selected k and the elbow reference shown in the panel are indicated by vertical dashed lines. (c) Average silhouette width across k = 2–10 for k-means clustering. The selected k and the k with the highest average silhouette shown in the panel are indicated by vertical dashed lines. of squares.

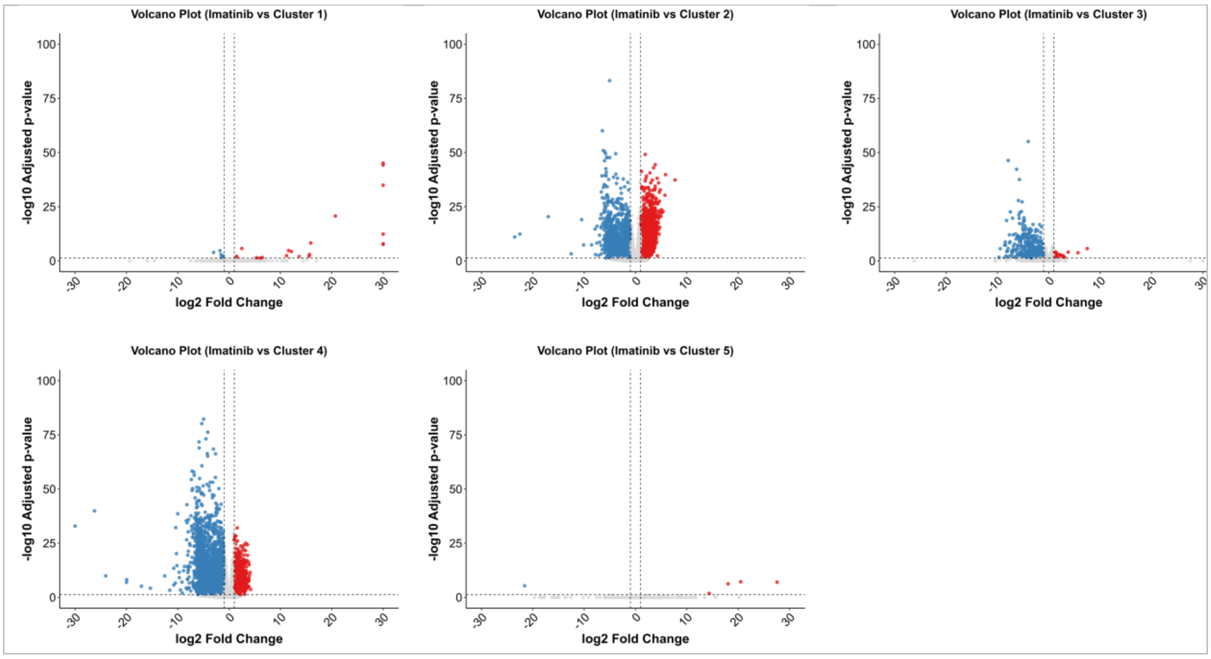

Figure S5. Cluster-specific differential expression of imatinib-treated samples in GSE186341.

Volcano plots show differential expression results for imatinib-treated samples compared with each of the five DMSO-defined control clusters (Clusters 1–5). Each point represents a gene, with the x-axis indicating $\log_2$ fold change and the y-axis indicating $-\log_{10}$ (adjusted p-value). Red and blue points denote significantly upregulated and downregulated genes, respectively, based on the adjusted p-value cutoff used in the analysis. Vertical dashed lines indicate the $\log_2$ fold-change cutoffs, and the horizontal dashed line indicates the adjusted p-value cutoff.

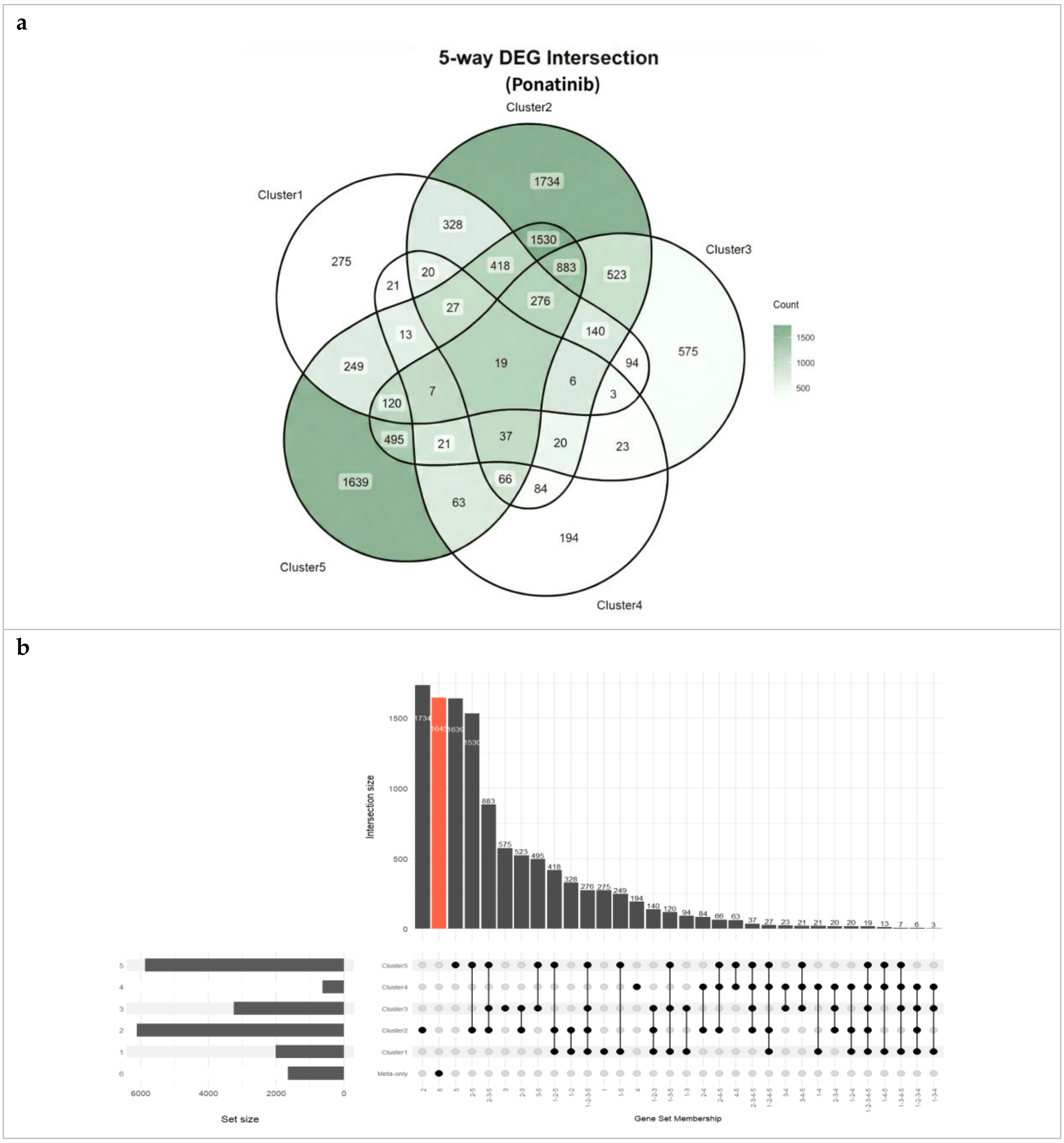

Figure S6. Intersections of cluster-wise DEGs in response to Ponatinib in GSE186341.

(a) Venn diagram showing overlap among DEGs identified from each of the five DMSO-defined control clusters (Clusters 1–5) using the same differential-expression criteria described in the Methods. Numbers indicate unique and shared genes across clusters. (b) UpSet plot summarizing unique and shared genes across combinations of the cluster-wise DEG sets. Bars indicate intersection sizes, and connected dots indicate the corresponding cluster combinations. The additional "Meta-only" set denotes genes that were identified as significant only at the meta-analysis level (i.e., not reaching significance in any single cluster-wise DEG list).

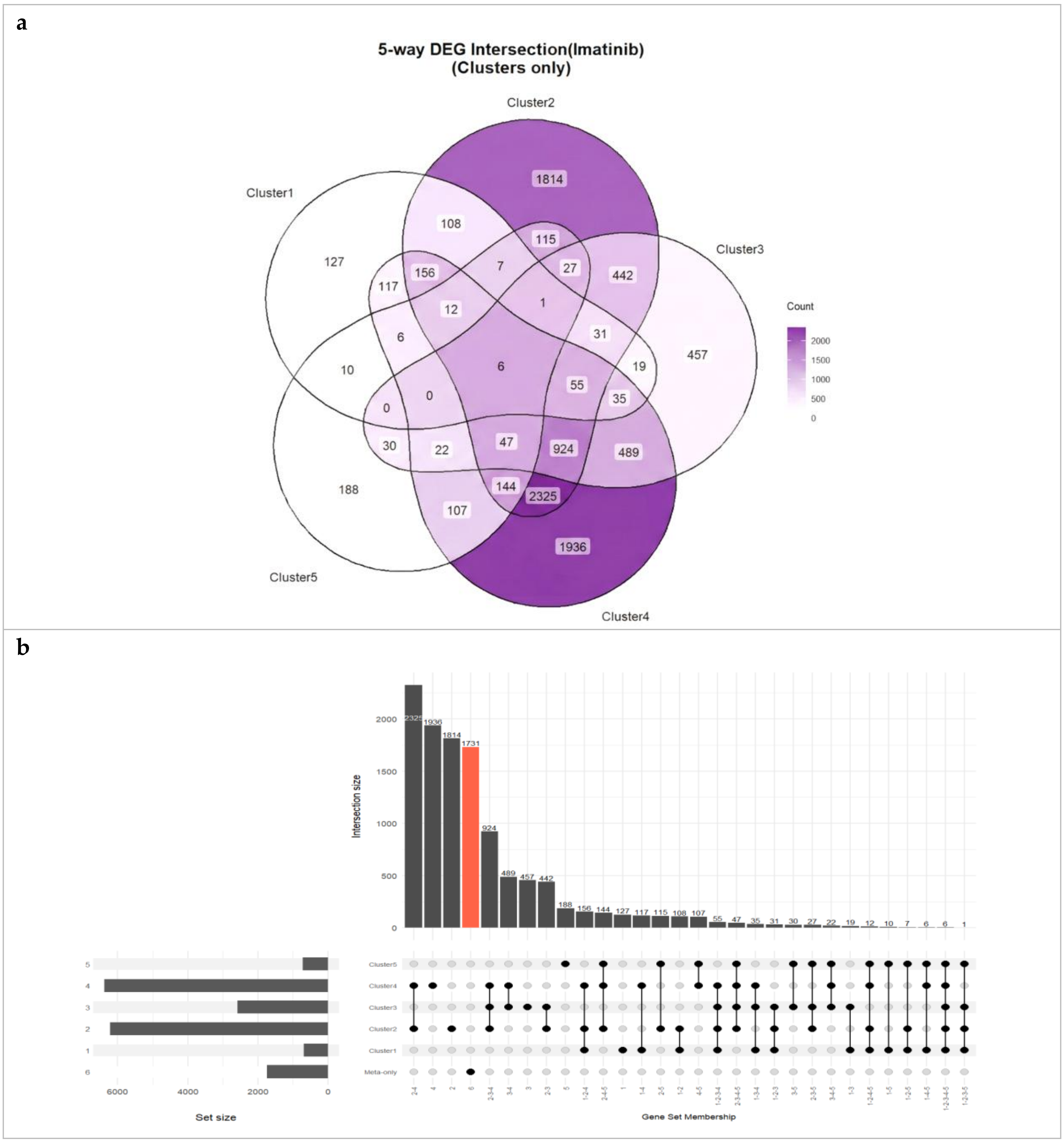

Figure S7. Intersections of cluster-wise DEGs in response to Imatinib in GSE186341.

(a) Venn diagram showing overlap among DEGs identified from each of the five DMSO-defined control clusters (Clusters 1–5) using the same differential-expression criteria described in the Methods. Numbers indicate unique and shared genes across clusters. (b) UpSet plot summarizing unique and shared genes across combinations of the cluster-wise DEG sets. Bars indicate intersection sizes, and connected dots indicate the corresponding cluster combinations. The additional “Meta-only” set denotes genes that were identified as significant only at the meta-analysis level (i.e., not reaching significance in any single cluster-wise DEG list).

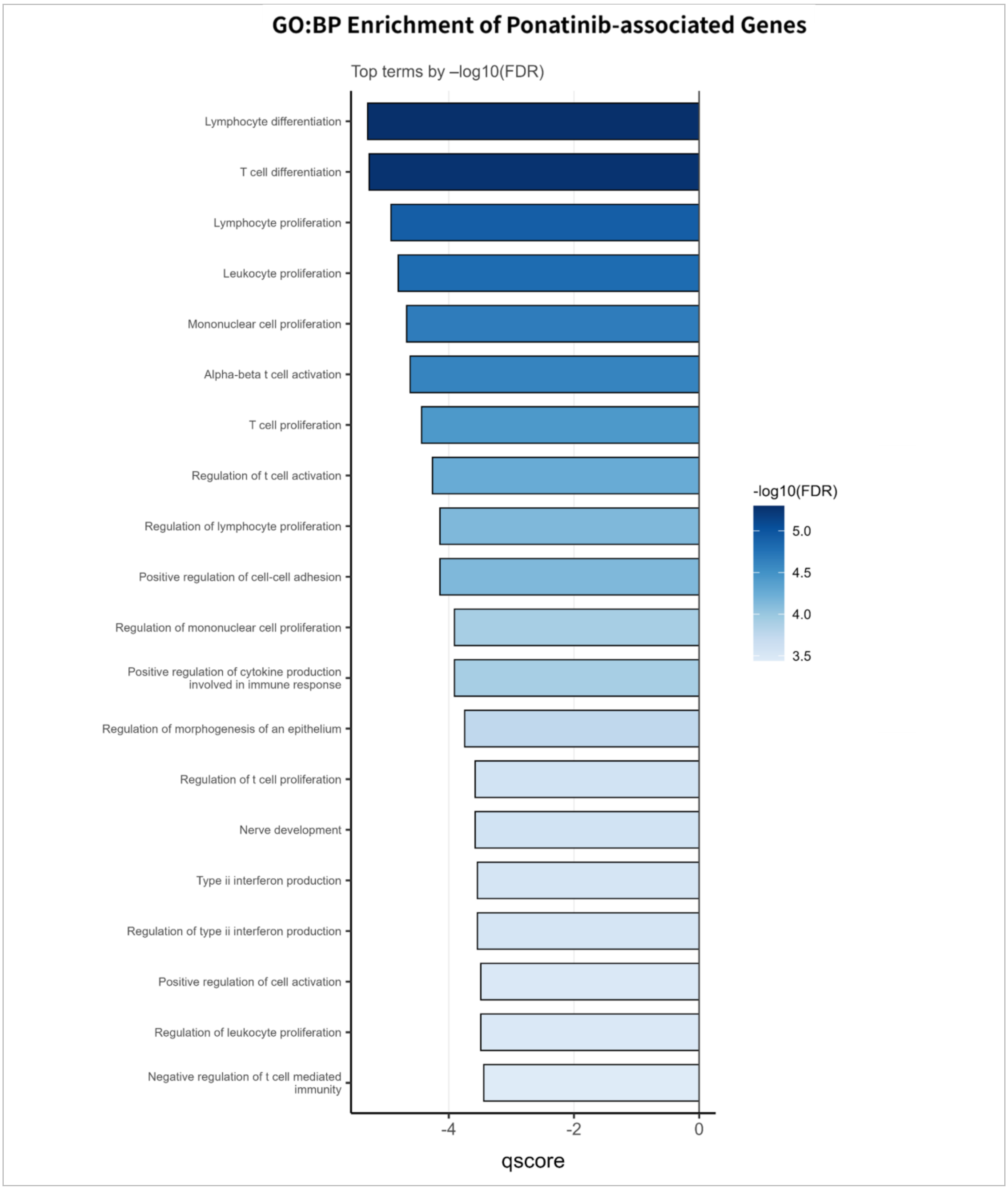

Figure S8. Direction-agnostic GO enrichment of Ponatinib-associated meta-significant genes in GSE186341.

Gene Ontology Biological Process (GO) enrichment was performed using the ponatinib-associated meta-significant gene set without separating up- and downregulated genes (i.e., direction-agnostic). Immune-related terms, including lymphocyte differentiation, T cell differentiation, and leukocyte/lymphocyte proliferation, were among the top enriched processes. Bar length indicates the q-score, and the color scale indicates enrichment significance ($-\log_{10}$ FDR).

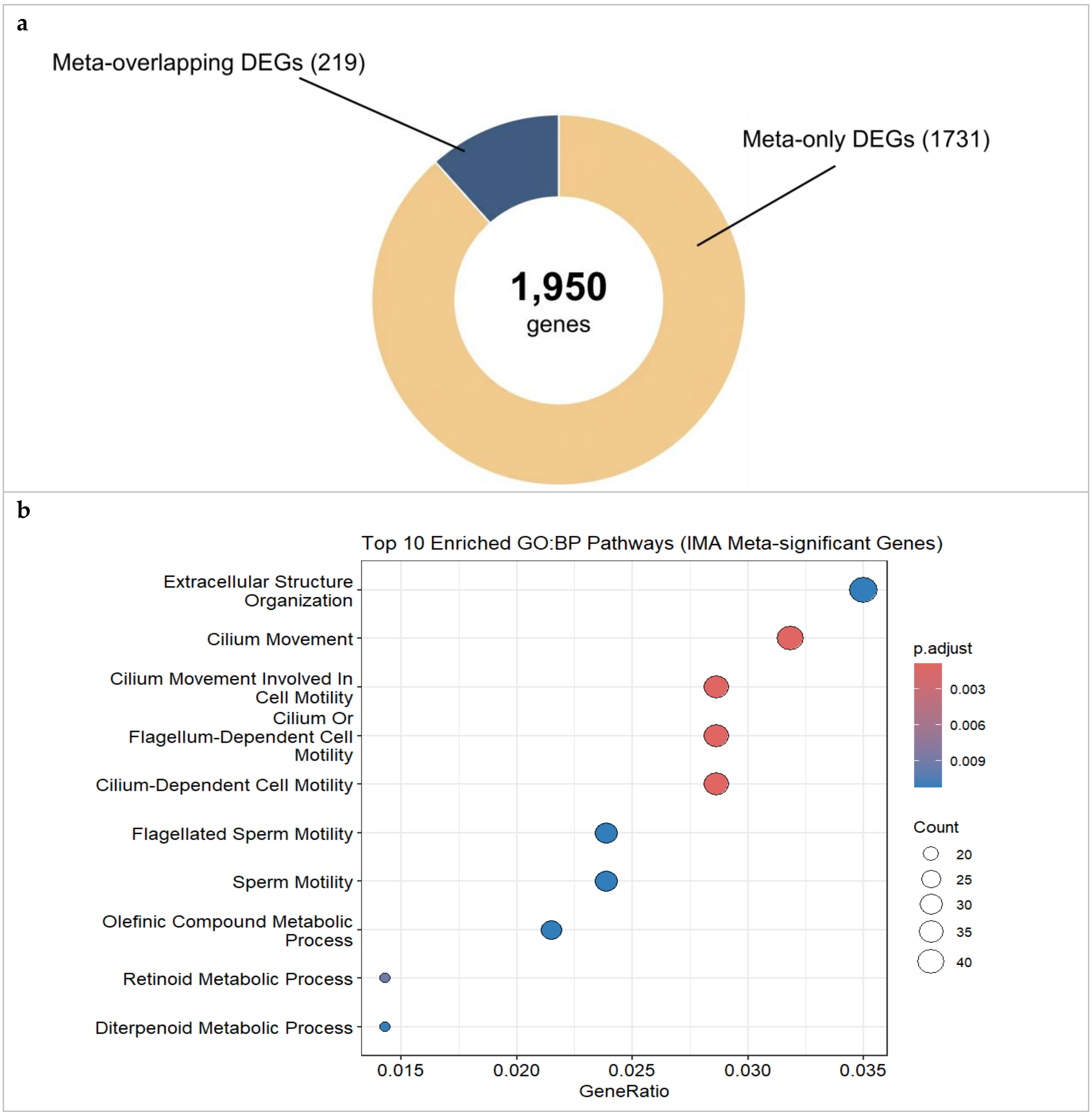

Figure S9. Meta-significant gene composition and enriched biological processes in response to Imatinib in GSE186341.

(a) Meta-analysis across the five DMSO-defined control clusters identified 1,731 meta-only DEGs and 219 meta-overlapping DEGs (total meta-significant genes, n = 1,950). Meta-only genes were significant only in the pooled analysis, meta-overlapping genes were significant in the pooled analysis and in at least one cluster-wise contrast, and pan-cluster genes were significant in all five cluster-wise contrasts. (b) GO enrichment analysis of meta-only DEGs highlighted terms related to cilium/flagellum-driven motility (e.g., cilium movement and cilium-dependent cell motility), sperm motility, and retinoid-related metabolic processes. Dot size indicates gene count per term, dot color indicates adjusted p value, and the x-axis indicates GeneRatio.

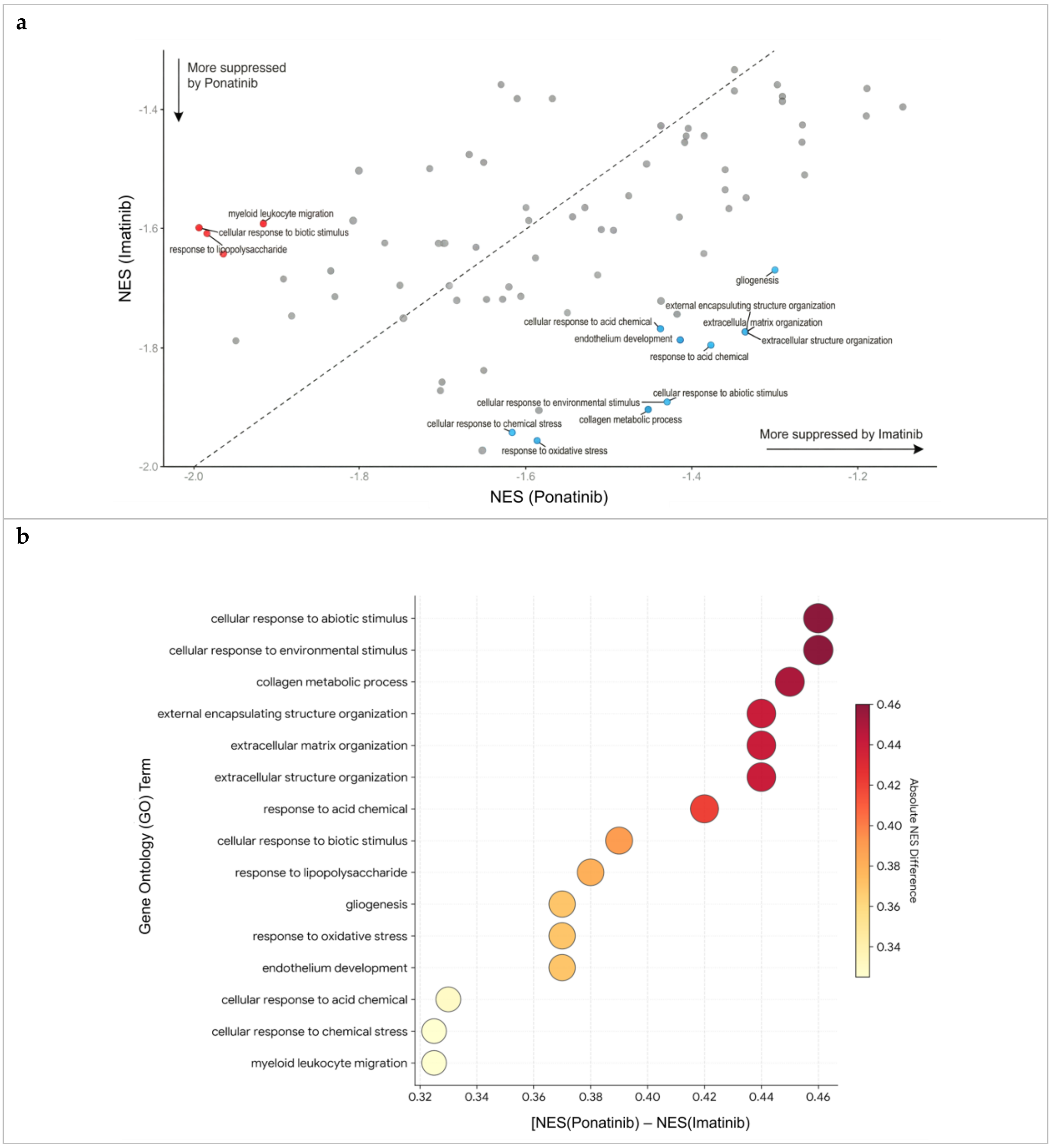

Figure S10. Comparative GSEA of GO terms between ponatinib and imatinib in GSE186341.

(a) Normalized enrichment scores (NES) for GO terms are compared between ponatinib- and imatinib-associated meta-significant gene sets. Each dot represents one GO term, and the dashed diagonal line indicates equal NES between the two drugs; colored points denote annotated terms, whereas gray points indicate other terms. (b) The top 15 GO terms with the largest absolute NES differences (|NES_PON – NES_IMA|) are shown. The x-axis indicates the absolute NES difference, and point color reflects |NES_PON – NES_IMA|.

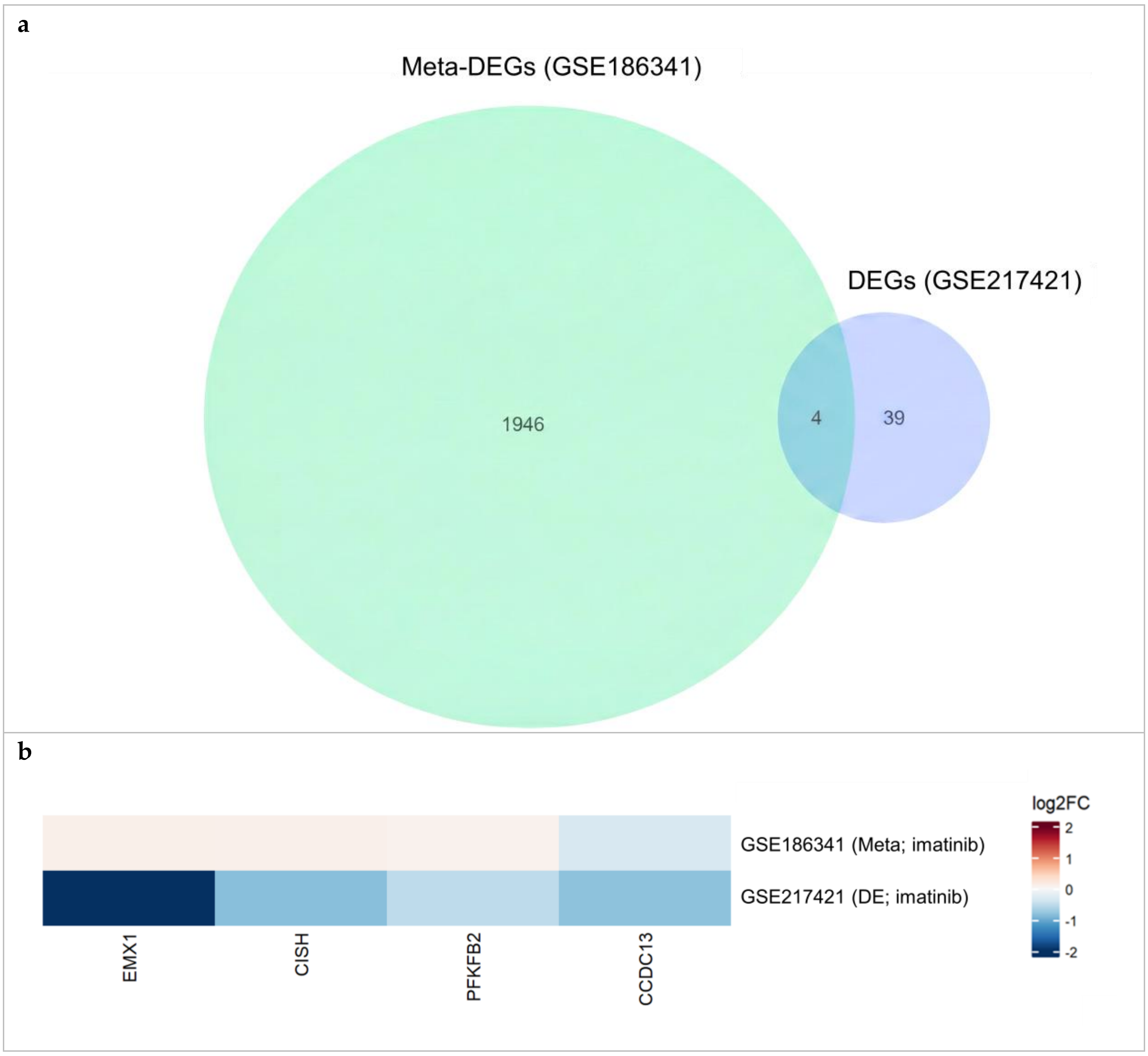

Figure S11. Cross-dataset overlap of the imatinib-associated gene signature between GSE186341 and GSE217421.

(a) Venn diagram showing the overlap between imatinib-associated meta-significant genes identified in GSE186341 and imatinib differentially expressed genes (DEGs) in the independent dataset GSE217421; four genes were shared. (b) Heatmap of $\log_2$ fold-change values for the four overlapping genes in each dataset.

Supplementary Tables

Table S1. Metrics supporting the selection of *k* for k-means clustering of DMSO controls in GSE186341.

k	WCSS	Silhouette (avg)	#Clusters	Size (min/med/max)	*n* threshold	Jaccard (mean)	Pred. strength	PAC
2	751296.1	0.050	2	60 / 232 / 404	15	0.506	0.990	0.000
3	533437.7	0.084	3	60 / 72 / 332	15	0.305	0.950	0.000
4	409388.2	0.117	4	60 / 114 / 176	15	0.214	0.604	0.341
**5**	**296130.8**	**0.154**	**5**	**60 / 60 / 212**	**15**	**0.181**	**0.768**	**0.091**
6	205095.9	0.189	6	60 / 60 / 152	15	0.185	0.897	0.000
7	149734.6	0.220	7	57 / 60 / 95	15	0.142	0.590	0.047
8	97798.9	0.256	8	48 / 60 / 72	15	0.119	0.767	0.019
9	91701.6	0.247	9	22 / 56 / 72	15	0.095	0.715	0.020
10	64553.2	0.275	10	22 / 48 / 72	15	0.085	0.632	0.023

Clustering was performed for $k = 2$–10 in the PC1–PC20 subspace. For each k, we report within-cluster sum of squares (WCSS), average silhouette width, bootstrap-based mean Jaccard similarity (cluster membership stability under resampling), prediction strength (stability under repeated two-fold splits), and the proportion of ambiguous clustering (PAC) derived from the consensus matrix using the (0.1, 0.9) interval (lower values indicate less ambiguity). Cluster-size summaries are shown as minimum/median/maximum, and n denotes the minimum cluster-size threshold used for stability calculations. The selected solution ($k = 5$) is highlighted.

Table S2. Cluster size distributions across k-means solutions for PCA-derived subgroups of GSE186341 DMSO controls.

k	Cluster sizes	Min *n*	Max *n*	Median *n*
2	60, 404	60	404	232
3	72, 332, 60	60	332	72
4	72, 156, 60, 176	60	176	114
5	60, 72, 60, 212, 60	60	212	60
6	60, 60, 152, 60, 60, 72	60	152	60
7	72, 60, 60, 60, 95, 60, 57	57	95	60
8	60, 72, 60, 56, 60, 48, 60, 48	48	72	60
9	60, 60, 48, 60, 48, 22, 56, 38, 72	22	72	56
10	22, 38, 72, 28, 60, 48, 60, 60, 28, 48	22	72	48

Sample counts per cluster are reported for each k-means solution (k = 2–10) derived from PCA of GSE186341 DMSO-treated samples. For each k, cluster sizes are listed together with the minimum, maximum, and median cluster size. For $k \geq 9$, the minimum cluster size decreased to n = 22–28.

Table S3. Nineteen genes shared across all five DMSO-defined subgroups in GSE186341.

Symbol	Gene name	Direction (in all 5)	Cluster1 padj	Cluster2 padj	Cluster3 padj	Cluster4 padj	Cluster5 padj
AAMDC	adipogenesis associated Mth938 domain containing	↓	6.85×10^{-3}	6.05×10^{-3}	2.66×10^{-2}	1.66×10^{-2}	2.89×10^{-5}
ARPC1B	actin related protein 2/3 complex subunit 1B	↓	1.59×10^{-3}	1.97×10^{-16}	1.96×10^{-9}	1.42×10^{-2}	2.55×10^{-7}
ATAD2	ATPase family AAA domain containing 2	↓	2.14×10^{-2}	7.40×10^{-6}	9.91×10^{-8}	3.28×10^{-2}	1.00×10^{-3}
CALM1	calmodulin 1	↓	1.83×10^{-2}	3.53×10^{-2}	2.02×10^{-2}	1.91×10^{-2}	4.89×10^{-5}
COL9A3	collagen type IX alpha 3 chain	↓	3.70×10^{-2}	7.60×10^{-4}	4.50×10^{-15}	1.66×10^{-2}	2.40×10^{-3}
DDTL	D-dopachrome tautomerase like	↓	1.04×10^{-3}	2.13×10^{-4}	6.83×10^{-4}	2.28×10^{-2}	1.60×10^{-4}
EIF3G	eukaryotic translation initiation factor 3 subunit G	↓	1.61×10^{-2}	2.02×10^{-5}	2.01×10^{-2}	2.56×10^{-2}	1.03×10^{-6}
EIF3H	eukaryotic translation initiation factor 3 subunit H	↓	3.64×10^{-2}	7.71×10^{-6}	3.95×10^{-2}	4.89×10^{-2}	1.59×10^{-5}
GLIPR2	GLI pathogenesis related 2	↓	9.51×10^{-3}	8.19×10^{-35}	4.84×10^{-6}	7.64×10^{-3}	2.85×10^{-8}
HSPA9	heat shock protein family A (Hsp70) member 9	↓	2.84×10^{-2}	3.70×10^{-18}	2.18×10^{-5}	1.69×10^{-2}	6.33×10^{-9}
LARP6	La ribonucleoprotein 6, translational regulator	↓	3.94×10^{-2}	2.82 ×	4.93 ×	7.32 ×	9.26 ×

				10^{-14}	10^{-2}	10^{-3}	10^{-27}
NTHL1	nth like DNA glycosylase 1	↓	8.81×10^{-4}	4.55×10^{-4}	4.90×10^{-9}	1.10×10^{-2}	3.15×10^{-2}
PHAF1	phagophore assembly factor 1	↓	4.77×10^{-4}	1.45×10^{-4}	4.09×10^{-2}	1.28×10^{-2}	2.18×10^{-3}
PKDCC	protein kinase domain containing, cytoplasmic	↓	1.84×10^{-2}	5.30×10^{-9}	2.31×10^{-2}	3.05×10^{-2}	1.20×10^{-16}
RTL10	retrotransposon Gag like 10	↓	3.73×10^{-4}	1.18×10^{-11}	1.36×10^{-8}	3.23×10^{-2}	5.67×10^{-3}
SEPTIN1	septin 1	↓	2.81×10^{-2}	1.48×10^{-6}	4.62×10^{-11}	1.61×10^{-2}	6.35×10^{-13}
SPPL2B	signal peptide peptidase like 2B	↓	8.09×10^{-8}	6.54×10^{-4}	2.83×10^{-4}	5.43×10^{-3}	9.57×10^{-3}
TRIP6	thyroid hormone receptor interactor 6	↓	1.52×10^{-3}	1.40×10^{-4}	4.60×10^{-2}	3.36×10^{-2}	5.24×10^{-4}
WNT7B	Wnt family member 7B	↓	1.95×10^{-2}	2.98×10^{-12}	2.40×10^{-3}	3.01×10^{-2}	5.82×10^{-29}

Genes were identified as the five-way intersection of subgroup-specific DEG lists (padj < 0.05 and $|\log_2 \text{FoldChange}| > 3$). Adjusted p values for each subgroup-specific contrast are shown. Direction is indicated relative to ponatinib after sign inversion of the original DESeq2 $\log_2$FoldChange values; all 19 genes showed concordant downregulation (↓) across the five subgroup-specific contrasts.

Table S4. Summary of differential expression and meta-analysis results for Ponatinib and Imatinib.

Drug	Total DEGs (FDR < 0.05)	Meta-significant genes	Meta-only genes	Cross-dataset overlap (GSE217421)
Ponatinib	215 (162)	2,639	1,645	81
Imatinib	207 (197)	1,950	1,731	4

Total DEGs (FDR < 0.05) from GSE186341, the number of meta-significant genes obtained by fixed-effect meta-analysis across the five DMSO-derived control clusters, the number of meta-only genes (significant only in the pooled meta-analysis), and the number of overlapping genes with the independent dataset GSE217421 are summarized for each drug. Values in parentheses indicate the subset with $|\log_2 FC| > 1$ in the single-contrast DEG results; this fold-change threshold was not applied in the meta-analysis.

Table S5. Cross-dataset comparison of 81 overlapping ponatinib-associated genes between GSE186341 and GSE217421.

Gene	Meta log_2FC (GSE186341)	log_2FC (GSE217421)	Adj. P (GSE217421)	Direction	$|\Delta log_2FC|$
KCNQ3	1.7496	0.6656	0.034	↑	1.08
SRPX2	1.6964	-1.4957	0.047	Discordant	3.19
FOXO1	1.0987	0.3579	0.006	↑	0.74
BDNF	0.6285	-0.8273	0.023	Discordant	1.46
ATP8B3	0.6108	0.8512	3.25 × 10^-6	↑	0.24
RAPGEF3	0.5662	0.5458	0.004	↑	0.02
TCAP	0.5378	-0.7573	0.001	Discordant	1.30
PRSS8	0.5280	-0.9182	0.008	Discordant	1.45
LFNG	0.5201	-1.4218	0.027	Discordant	1.94
ZNF185	0.4898	0.2241	0.009	↑	0.27
FBXO43	0.4748	0.8263	0.002	↑	0.35
PTX3	0.4710	-1.2734	0.031	Discordant	1.74
BORA	0.4544	0.6626	6.17 × 10^-4	↑	0.21
CENPI	0.4357	0.4020	0.030	↑	0.03
LAD1	0.3390	-0.6298	0.027	Discordant	0.97
FAM43A	0.3353	-0.6254	0.018	Discordant	0.96
GPLD1	0.3233	0.3677	0.045	↑	0.04
CRYM	0.3111	0.7881	0.037	↑	0.48
IRS1	0.2575	0.5908	7.35 × 10^-4	↑	0.33
KCNN3	0.2514	0.6984	0.023	↑	0.45
MELTF	0.2188	-0.9930	5.95 × 10^-4	Discordant	1.21
DNAAF3	0.1976	-0.9129	0.009	Discordant	1.11
HEATR4	0.1866	0.5953	0.020	↑	0.41
PLEKHA4	0.1648	-0.5673	2.18 × 10^-5	Discordant	0.73
RUSC2	0.1394	-0.3694	0.014	Discordant	0.51
KLHL3	0.1357	0.3959	0.012	↑	0.26
C3orf52	0.1260	0.5203	1.27 × 10^-4	↑	0.39
TEAD3	0.1187	-0.3272	0.006	Discordant	0.45
HYLS1	0.1174	0.4818	4.92 × 10^-5	↑	0.36
ALDH5A1	0.1082	0.2487	0.020	↑	0.14
KIAA0586	0.0331	0.2887	0.009	↑	0.26
SCN3B	0.0312	-0.7591	0.024	Discordant	0.79
KCNJ2	0.0197	0.6882	0.001	↑	0.67
GALM	-0.0218	0.2817	0.002	Discordant	0.30
RPS6KA1	-0.0290	-0.3728	0.041	↓	0.34
HECW2	-0.0345	-0.3623	0.040	↓	0.33
CASP6	-0.0407	0.1575	0.044	Discordant	0.20
OPHN1	-0.0514	-0.4083	0.049	↓	0.36
MCL1	-0.1167	-0.3584	0.008	↓	0.24
MXD3	-0.1222	0.6626	5.31 × 10^-4	Discordant	0.78
ANKRD33B	-0.1279	-0.7463	0.008	↓	0.62
IL1RAP	-0.1342	-0.5351	0.043	↓	0.40
MTCL1	-0.1464	-0.5000	0.016	↓	0.35
SGMS2	-0.1553	-0.5616	0.016	↓	0.41
PLA2G15	-0.1572	-0.2434	0.016	↓	0.09

ALDH2	-0.2263	0.2937	0.008	Discordant	0.52
ADAM22	-0.2494	0.5249	0.003	Discordant	0.77
QPCT	-0.3190	-0.6766	0.033	↓	0.36
EREG	-0.3477	-0.9305	0.027	↓	0.58
GCH1	-0.3481	-0.5914	0.016	↓	0.24
KIAA0319	-0.3711	0.5642	0.037	Discordant	0.94
FAM185A	-0.4204	0.2610	0.025	Discordant	0.68
GBE1	-0.4209	-0.5137	0.004	↓	0.09
IFT81	-0.5023	0.3393	9.04 × 10^-4	Discordant	0.84
LTBP1	-0.5217	-0.2341	0.040	↓	0.29
ESAM	-0.5323	-1.6715	0.018	↓	1.14
KCTD18	-0.5330	0.1815	0.039	Discordant	0.71
HMGCS1	-0.5339	-0.3832	0.016	↓	0.15
SYCE2	-0.5483	0.4456	0.020	Discordant	0.99
UBQLN4	-0.6213	-0.1722	0.037	↓	0.45
JDP2	-0.6330	0.6503	0.038	Discordant	1.28
HIRA	-0.6564	-0.2424	0.008	↓	0.41
ATG9A	-0.7107	-0.2325	0.006	↓	0.48
IL7R	-0.7111	-2.3507	0.025	↓	1.64
NPHP1	-0.7485	0.2897	0.019	Discordant	1.04
HK2	-0.8282	-0.5630	0.021	↓	0.27
CC2D2A	-0.8325	0.3544	9.16 × 10^-5	Discordant	1.19
ACTA1	-0.8398	-1.5826	6.61 × 10^-5	↓	0.74
ZNF30	-0.8539	0.4273	0.005	Discordant	1.28
TMEM217	-0.8697	-0.7110	0.028	↓	0.16
NES	-0.8854	-0.6336	0.036	↓	0.25
KRBA1	-0.9174	-0.8570	2.46 × 10^-8	↓	0.06
WNT9A	-0.9272	-0.6796	0.004	↓	0.25
NRTN	-0.9904	-0.5387	0.023	↓	0.45
MAP3K7CL	-1.0277	-1.3311	6.17 × 10^-4	↓	0.30
KLHDC9	-1.0319	0.4420	0.026	Discordant	1.47
SPTSSB	-1.2965	1.2197	0.042	Discordant	2.52
SPOCK2	-1.3229	-1.0010	0.015	↓	0.32
KLHDC8B	-1.4878	-0.4488	0.002	↓	1.04
GREB1	-1.6984	0.4182	0.020	Discordant	2.12
RTKN2	-1.7214	0.6289	0.008	Discordant	2.35

The table lists the 81 genes overlapping between the ponatinib-associated meta-signature from GSE186341 and the differential expression analysis of GSE217421. Meta log_2FC denotes the pooled meta-analytic log_2 fold-change estimate from GSE186341, and log_2FC (GSE217421) denotes the corresponding fold-change estimate from GSE217421. Adj. P (GSE217421) denotes the Benjamini–Hochberg adjusted P value from GSE217421. Direction denotes concordant downregulation, concordant upregulation, or discordant direction between datasets. $|\Delta log_2FC|$ denotes the absolute difference between the meta log_2FC from GSE186341 and the log_2FC from GSE217421. Genes are grouped as concordant down, concordant up, and discordant, and are ordered by adjusted P value from GSE217421 within each group.

Table S6. Cluster-specific and cross-dataset log_2 fold-change estimates for six representative ponatinib-associated genes.

Gene	Cluster1	Cluster2	Cluster3	Cluster4	Cluster5	Meta log_2FC (GSE186341)	log_2FC (GSE217421)	Direction
MCL1	1.139	-0.147	0.182	0.258	-0.351	-0.117	-0.358	Negative
GCH1	-1.481	-3.608	-0.645	2.074	0.988	-0.348	-0.591	Negative
HK2	-2.216	-1.405	0.929	-0.712	-1.274	-0.828	-0.563	Negative
RAPGEF3	-4.417	1.908	-0.223	0.028	-3.181	0.566	0.546	Positive
FOXO1	-2.081	1.841	-1.097	-1.399	1.23	1.099	0.358	Positive
KCNN3	-5.849	-5.137	-1.022	-0.942	0.897	0.251	0.698	Positive

Six representative genes selected for Figure 7c are shown. Cluster1–Cluster5 denote cluster-specific log_2 fold-change estimates from GSE186341, followed by the pooled meta-analytic log_2 fold-change estimate for GSE186341 and the corresponding log_2 fold-change estimate from GSE217421. Direction denotes concordant downregulation or concordant upregulation across GSE186341 and GSE217421.

Table S7. Summary of statistical criteria used for transcript-level candidate selection.

Analysis step	Statistical criteria
1. Cluster-wise DESeq2	design = ~ PC1 + PC2 + PC3 + treatment; FDR < 0.05
2. Fixed-effect meta-analysis	available in ≥ 2 contrasts; directional consistency in ≥ 4 clusters; I^2 < 50%; two-sided meta-analysis p < 0.05
3. Validation DESeq2	GSE217421; padj < 0.05
4. Imatinib comparison	same analytic pipeline applied to imatinib; genes meeting ponatinib but not imatinib criteria classified as ponatinib-specific
5. Cross-dataset overlap	intersection of GSE186341 meta-DEGs and GSE217421 DEGs

Cluster-wise differential expression was assessed in GSE186341 by DESeq2, followed by inverse-variance weighted fixed-effect meta-analysis across clusters. Validation-stage differential expression was assessed in GSE217421. Cross-dataset candidate selection was based on overlap between GSE186341 meta-DEGs and GSE217421 DEGs, with parallel imatinib analysis used for comparative classification of ponatinib-specific candidates.
